# Quartet protein reference materials and datasets for multi-platform assessment of label-free proteomics

**DOI:** 10.1186/s13059-023-03048-y

**Published:** 2023-09-07

**Authors:** Sha Tian, Dongdong Zhan, Ying Yu, Yunzhi Wang, Mingwei Liu, Subei Tan, Yan Li, Lei Song, Zhaoyu Qin, Xianju Li, Yang Liu, Yao Li, Shuhui Ji, Shanshan Wang, Proteomic Massive Analysis and Quality Control Consortium, Zhaoyu Qin, Qingyu He, Xingfeng Yin, Lunzhi Dai, Haiteng Deng, Chao Peng, Ping Wu, Minjia Tan, Jing Jiang, Yaoyang Zhang, Yunxia Li, Wenqin Liu, Wei Chen, Rui Wang, Jin Zi, Qidan Li, Mingzhou Bai, Zeng Wang, Zhanlong Mei, Zhongyi Cheng, Jun Zhu, Xuemei Wu, Xing Yang, Yue Zhou, Yuanting Zheng, Fuchu He, Jun Qin, Chen Ding

**Affiliations:** 1 State Key Laboratory of Genetic Engineering and Collaborative Innovation Center for Genetics and Development, School of Life Sciences, Institutes of Biomedical Sciences, Human Phenome Institute, Zhongshan Hospital, Fudan University, Shanghai, 200433 China; 2 State Key Laboratory of Proteomics, Beijing Proteome Research Center, National Center for Protein Sciences (Beijing), Beijing Institute of Lifeomics, Beijing, 102206 China

**Keywords:** Quantitative proteomics, Reference materials, Benchmark datasets, Reproducibility, Injection order

## Abstract

**Background:**

Quantitative proteomics is an indispensable tool in life science research. However, there is a lack of reference materials for evaluating the reproducibility of label-free liquid chromatography-tandem mass spectrometry (LC–MS/MS)-based measurements among different instruments and laboratories.

**Results:**

Here, we develop the Quartet standard as a proteome reference material with built-in truths, and distribute the same aliquots to 15 laboratories with nine conventional LC–MS/MS platforms across six cities in China. Relative abundance of over 12,000 proteins on 816 mass spectrometry files are obtained and compared for reproducibility among the instruments and laboratories to ultimately generate proteomics benchmark datasets. There is a wide dynamic range of proteomes spanning about 7 orders of magnitude, and the injection order has marked effects on quantitative instead of qualitative characteristics.

**Conclusion:**

Overall, the Quartet offers valuable standard materials and data resources for improving the quality control of proteomic analyses as well as the reproducibility and reliability of research findings.

**Supplementary Information:**

The online version contains supplementary material available at 10.1186/s13059-023-03048-y.

## Background

The launch of the Human Phenome Project [1–4] and the Human Proteome Project [5–9] has become the consensus of the scientific community in the post-genomic era and brought the field of proteomics to a prominent position in the life sciences owing to its implications in precision medicine. Continuous improvements in next-generation proteomics, such as in instrumentation, sample preparation, and computational analysis, have facilitated the generation of large amounts of data, including protein profiles, post-translational modifications, and protein–protein interactions [10–13]. Similar to other omics technologies (e.g., genomics and transcriptomics), low reproducibility across large-scale experiments remains one of the most critical challenges in proteomics [14, 15]. This poor reproducibility is mainly attributable to the large variability introduced by heterogeneity in experimental design, sample processing methods, environmental and operating conditions, mass spectrometers, data search algorithms, statistical analysis methods, and other conditions across studies and laboratories [16].

The US Food and Drug Administration (FDA)-led Microarray and Sequencing Quality Control (MAQC/SEQC) consortium conducted three phases of projects to assess the reliability and reproducibility of genomics technologies, including microarrays, genome-wide association studies, and next-generation sequencing [17–19]. At the end of 2017, the MAQC consortium announced the formation of the MAQC Society, an international society dedicated to the quality control and analysis of massive data generated from high-throughput technologies with the goal of enhancing reproducibility [20]. In addition, the Microbiome Quality Control (MBQC) project was established to evaluate and, ultimately, standardize measurement methods for the human microbiome and includes protocols for handling human microbiome samples and computational pipelines for microbial data processing [21].

Similarly, tremendous effort has been made to acquire high-quality and reproducible data in the field of proteomics. These efforts include the production of yeast standard [22], the centralized analysis for determining sources of irreproducibility in LC–MS/MS-based proteomics [23], the use of protein mixtures for repeatability and reproducibility measurements [24], longitudinal quality assessment [25], the generation of reference xenograft proteomes for evaluating variability in differentially expression analysis [26], the assessment of reproducibility using SWATH-MS data acquisition mode [27], and the establishment of a quality control framework [28], etc. These efforts have improved the accessibility of quality control in biological mass spectrometry and have the following characteristics: (a) Most of them distributed the aliquots from a single sample to multiple laboratories for proteome identification, which met some benchmarking and QC needs; (b) The pairwise comparisons between samples have also been made for initial platform validation and ongoing quality control on multiple instruments across different laboratories; (c) Mass spectrometry instruments mostly launched before the year 2015, including the LTQ and Q-Exactive, were evaluated.

As an extension of previous studies, we initiated the proteome QC project as part of MAQC phase IV (MAQC-IV). In this study, we developed the Chinese Quartet (hereafter referred to as “the Quartet”) as a proteome standard comprising four standard samples derived from four members of the same family. For establishing the Quartet, intrinsic differences between samples were defined as “built-in truths,” which enabled evaluation of the qualitative and quantitative characteristics of LC–MS/MS-derived data. We compared the proteins identified in all measurements of each sample of the Quartet and identified differentially expressed proteins (DEPs) across experiments with three technical replicates. The performance of different platforms was quantitatively evaluated using the signal-to-noise ratio (SNR) to determine variations and differences among samples. We further evaluated batch effects due to differences in the injection order of LC–MS/MS and performed absolute quantification of the Quartet standards using C^13^ stable isotope-labeled concatenated peptides (QconCAT). As a result, we provide a standard reference material for evaluating the reproducibility and reliability of different MS-based proteomics strategies. The variations introduced by different factors, including MS principles, instrument specs, and temporal and spatial issues, provide a valuable resource for the development and optimization of proteomics SOPs that are applicable to both basic and clinical studies [29].

## Results

### Establishment of the Quartet

**Fig. 1:**
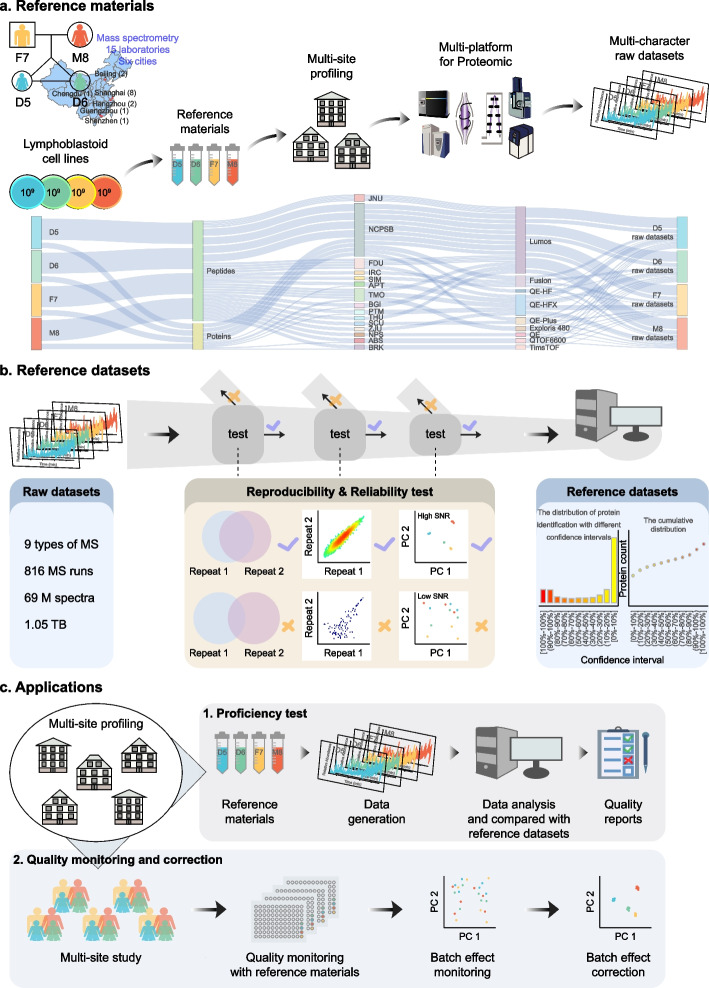
Study design and implementation. **A** Lymphoblastoid cells provided by a family, consisting of a father (F7, yellow), mother (M8, red), and a pair of twin daughters (D5 and D6, apple green and azure), were used to prepare the proteome reference materials (i.e., the Quartet) in both protein and peptide forms. The aliquots were sent to 15 laboratories, and proteomics analyses by data-dependent acquisition (DDA) were carried out using Orbitrap- and TOF-based systems. All LC–MS/MS files were aggregated at Fudan University for analysis. **B** A total of 816 MS files were used to qualitatively and quantitatively evaluate the reproducibility and reliability of the proteome. Furthermore, reference protein sets with confidence intervals were determined. **C** Application scenarios of the reference materials

Lymphoblastoid cells provided by a Chinese family from the Fudan University (FDU) Taizhou cohort [30–32], including the father (F7), mother (M8), and a pair of twin daughters (D5 and D6), were used to establish immortalized cell lines. Protein extraction from cells and peptide digestion were uniformly performed in the National Center for Protein Sciences (NCPSB, Beijing, China) laboratory. We distributed these aliquots to 15 laboratories throughout six cities in China (Fig. 1A). Nine types of conventional LC–MS/MS platforms, including Orbitrap-based systems (e.g., Orbitrap Lumos and Q-Exactive) or time-of-flight (TOF)-based systems (TripleTOF 6600 and timsTOF pro), were incorporated in the proteomics platform evaluation. All raw LC–MS/MS files were uploaded into Firmiana [33], and quantitative proteome matrix results were obtained following the standard computational workflow for proteomics data. The assessment for multi-character datasets was performed by the bioinformatics team at the FDU laboratory. The advantage of this strategy was that any factors influencing the identification and quantification of (differentially expressed) proteins in proteomics data were restricted to the different sample types, sites, and instruments, excluding any variations in sample preparation and downstream bioinformatics methods.

All measurements resulted in a global dataset containing 816 MS files generated from the 15 laboratories (Additional file 2: Table S1), providing a rich resource for performing both qualitative and quantitative reproducibility and reliability assessments while promoting the clinical translation and application of proteomics. Among the files, 792 MS files were used for the following purposes: cross-platform assessment with a single-shot proteomics strategy, which acquired a total of 288 LC–MS/MS runs (4 samples × 3 replicates × 24 experiments) using the Quartet standards at the peptide level (Additional file 1: Fig. S1 A); deep coverage with a sample fractionation strategy, which produced a total of 384 LC–MS/MS runs using the Quartet standards at the protein level (Additional file 1: Fig. S1 B); and longitudinal proteomic monitoring for 15 months, which was used for sample stability evaluation, generating 120 MS files using the Quartet standards at both peptide and protein levels (Additional file 1: Fig. S1 C).

The reference material (i.e., the Quartet) allowed us to perform daily QC tests. Using the entire dataset, we further identified reference protein sets with confidence intervals to provide important benchmarks for the application of LC–MS/MS technologies to clinical assays. The confidence intervals were determined according to the frequency of protein occurrence in all experiments. These reference datasets can offer a comparable baseline for guiding users to carry out proficiency tests, promoting parameter optimization and method development. The Quartet can also be used to assess the reproducibility and performance of large-scale quantitative proteomics among different instruments and laboratories. The built-in individual differences can facilitate evaluation of the reproducibility of differential proteome platforms (Fig. 1B, C).

### Characteristics of the Quartet

**Fig. 2:**
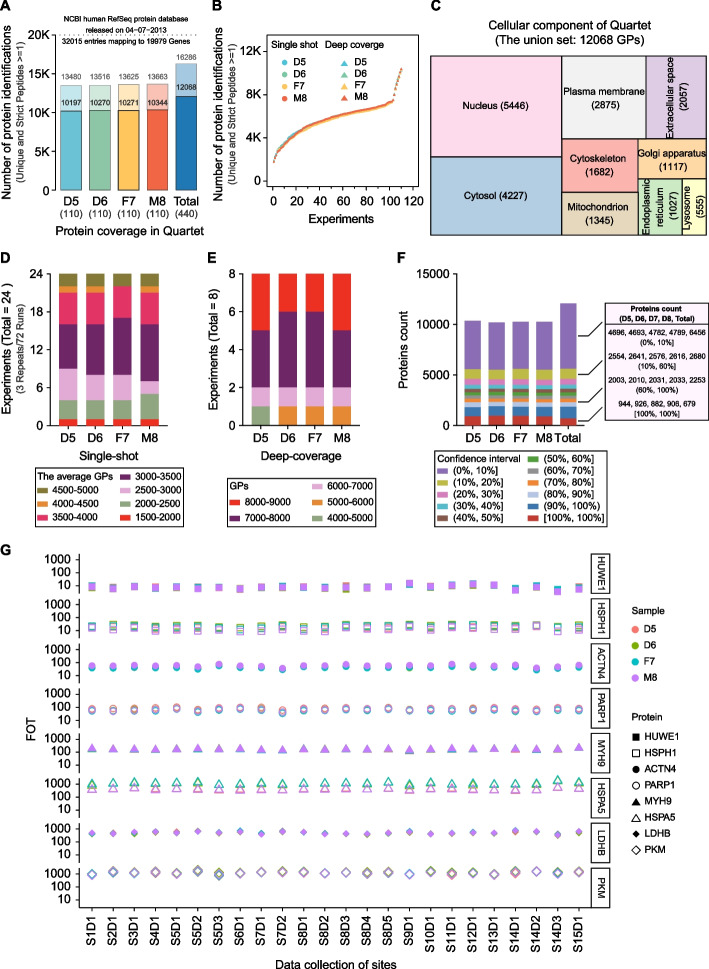
Characteristics of the Quartet. **A** Proteins identified from the Quartet samples. **B** Cumulative number of proteins identified as a function of experiment numbers. **C** Gene ontology analysis of cellular component terms (GO-CC). **D** Average number of proteins identified from different experiments using the single-shot strategy. **E** Number of proteins identified from different experiments using a deep-coverage strategy. **F** Number of proteins identified distributed over 11 confidence intervals. The stack column labeled “Total” indicates the global distribution of all proteins identified from the Quartet samples. Right panel: detailed protein identification information within rough confidence intervals: 0–10%, 10–60%, 60–100%, and 100–100%. **G** Proteins with high quantitative stability distributed into different quantitative orders of magnitude

To delineate the proteomic portraits of the reference materials, we performed 110 MS runs on each sample, including 102 single-shot and 8 deep-coverage MS runs. Over 10,000 proteins with at least one unique and strict peptide were detected in all measurements of each Quartet sample. Up to 12,068 proteins were identified in the Quartet using state-of-the-art high-resolution MS, indicating that MS technology is capable of a coverage of 10,000 proteins per sample (Fig. 2A, B). The deep-coverage strategy with multiple fractions (≥ 6) detected ~ 4000 more proteins than did the single-shot strategy. Gene ontology enrichment analysis (http://geneontology.org) for cellular components of the Quartet proteome mainly resulted in nine terms, covering the nucleus, cytosol, plasma membrane, cytoskeleton, mitochondrion, extracellular space, Golgi apparatus, endoplasmic reticulum, and lysosome, demonstrating the complexity and diversity of the reference materials (Fig. 2C). In the 24 experiments (with three technical replicates) using the single-shot strategy, the average number of proteins identified in each sample varied from 1500 to 5000 depending on the different mass spectrometers used in the 15 test sites (Fig. 2D). For example, ~ 2000 proteins were detected with the Q Exactive and TripleTOF 6600 instruments, whereas over 4500 proteins were identified with the Exploris 480 and timsTOF Pro instruments. Notably, Site 11 identified less than 2000 proteins with Q Exactive Plus, which was highly inconsistent with the empirical value, suggesting that the state of the LC–MS/MS system should be inspected or that an inferior proteomics workflow was carried out. In the eight fractionation experiments, with 6, 12, 18, or 30 fractions, over 4500 proteins were identified in each sample, ranging from 4745 proteins identified with the TripleTOF 6600 system using six fractions to 8441 proteins identified with the Fusion Lumos system using 30 fractions (Fig. 2E).

We divided the proteins identified from 110 MS runs of each Quartet sample into 11 groups with different confidence intervals according to their occurrence frequency in all experiments (observation times/total measurement times). For each sample, proteins with a confidence interval of 0–10%, 10–60%, 60–100%, and 100–100% accounted for approximately 46, 25, 20, and 9% of the total, respectively. In the whole proteome (12,068 proteins) of the Quartet, 679 proteins (6%) with ultra-high confidence were identified in all 440 MS runs (Fig. 2F), including some proteins with high quantitative stability that were distributed into different quantitative levels (Fig. 2G), such as HUWE1, HSPH1, ACTN4, PARP1, MYH9, HSPA5, LDHB, and PKM, which have potential value as internal “anchor” proteins for quantification. During quantitative analysis of a complex clinical proteome, the calibration of protein profiles in the experiment according to these anchor proteins may improve the reliability of the detected protein abundance.

### Differential proteome as a reliability indicator

**Fig. 3:**
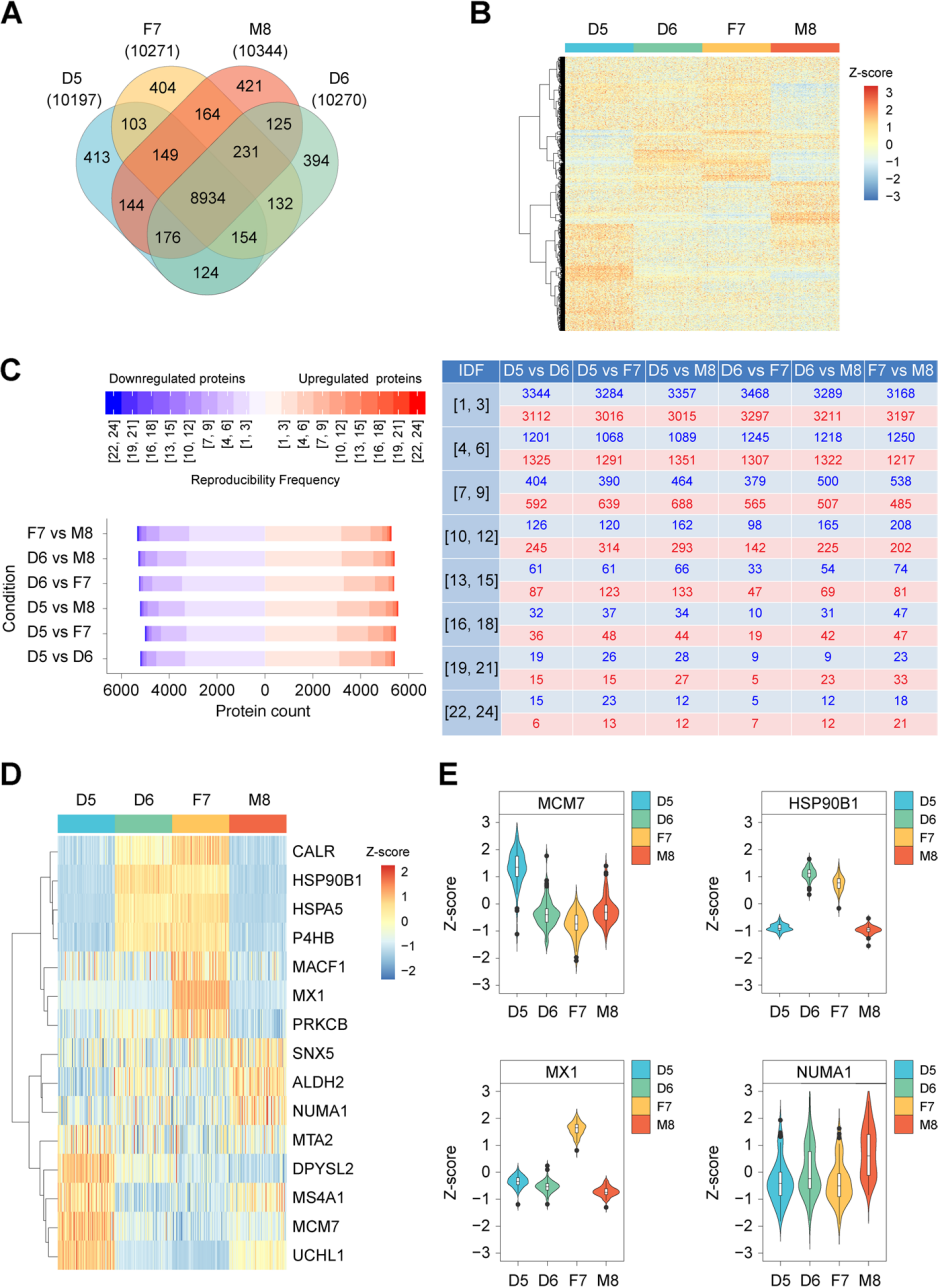
Differential proteome as a reliability indicator. **A** Venn diagram of proteins identified in the Quartet samples. **B** Heatmap of differentially expressed proteins. Cluster analysis was performed using *Z*-score-transformed protein intensities for the proteins with expression level fold-change > 2. Red indicates a high expression level and blue indicates a low expression level. **C** Occurrence frequency of differentially expressed proteins (DEPs), according to the pairwise comparisons between samples of the Quartet, over 24 experiments with three technical replicates each using a single-shot strategy. Left panel: number of DEPs in different partition intervals for each pairwise comparison. Red and blue indicate upregulation and downregulation directions of DEPs within a comparison, respectively, such as D5 versus D6, with D6 as the reference group. Right panel: numeric table corresponding to the distribution map in the left panel. IDF: identification frequency. **D** Heatmap of 15 DEPs with a consistent upregulation/downregulation trend across all 24 experiments. **E** Proteins with highly sample-specific expression in the Quartet

The Quartet was designed to include built-in truths among the samples of the four family members to discover quantitative differences across proteome platforms. In the whole Quartet proteome (12,068 proteins), 74% (8934) of the proteins were common to the D5, D6, F7, and M8 samples. In contrast, each sample contained ~ 4% specific proteins, ranging from 394 proteins in D6 to 421 proteins in M8 (Fig. 3A). Differential proteomics analysis was then performed for the 24 experiments (with three technical replicates) using the single-shot strategy. Using a fold-change threshold of > 2 observed at least once in pairwise comparisons of the four Quartet samples within a single experiment, a total of 1662 DEPs were identified across all experiments, demonstrating the sample-specific characteristics that can be used for constructing built-in truths of reference materials (Fig. 3B). Even though samples D5 and D6 had identical genomes, they exhibited differential gene expression patterns at the proteome level, reflecting the complexity and variations in the transcription and translation processes among individuals.

Furthermore, to determine the high-confidence DEPs that can serve as “indicators” to evaluate the quantitative reliabilities of different MS platforms, we counted the occurrence frequency of DEPs in the 24 experiments by pairwise comparisons between samples within each experiment, which was considered to reflect the reproducibility frequency of DEPs. We divided the reproducibility frequency of DEPs into eight intervals as shown in Fig. 3C. The results indicated 5418 upregulated proteins and 5202 downregulated proteins (DEP pool) in D5 compared with D6 in at least one experiment (Additional file 3: Table S2 and Additional file 4: Table S3), of which 94.0, 5.5, and 0.5% exhibited a low (1–9), medium (10–18), and high (19–24) reproducibility frequency, respectively. After applying the same analysis for other pairwise comparisons within the family, it became apparent that the main source of variation causing low reproducibility is likely different mass spectrometer models, different test sites, and random MS/MS sampling by data-dependent acquisition. DEPs with medium (10–18) and high (19–24) reproducibility frequencies showed high robustness, which generally reflected real differences between the samples in the Quartet. These proteins can be used as a benchmark dataset to evaluate the quantitative ability of comparative proteomics on different platforms. Additionally, we found 15 DEPs with consistent upregulation/downregulation trends in all 24 experiments (Fig. 3D); for example, MCM7, HSP90B1, MX1, and NUMA1 exhibited significantly high expression levels in D5, D6, F7, and M8, respectively (Fig. 3E). Overall, the detection rates and differential expression patterns of these proteins can serve as a baseline for evaluating the performance of different LC–MS/MS platforms in proteome detection.

### Variation among MS instruments

**Fig. 4:**
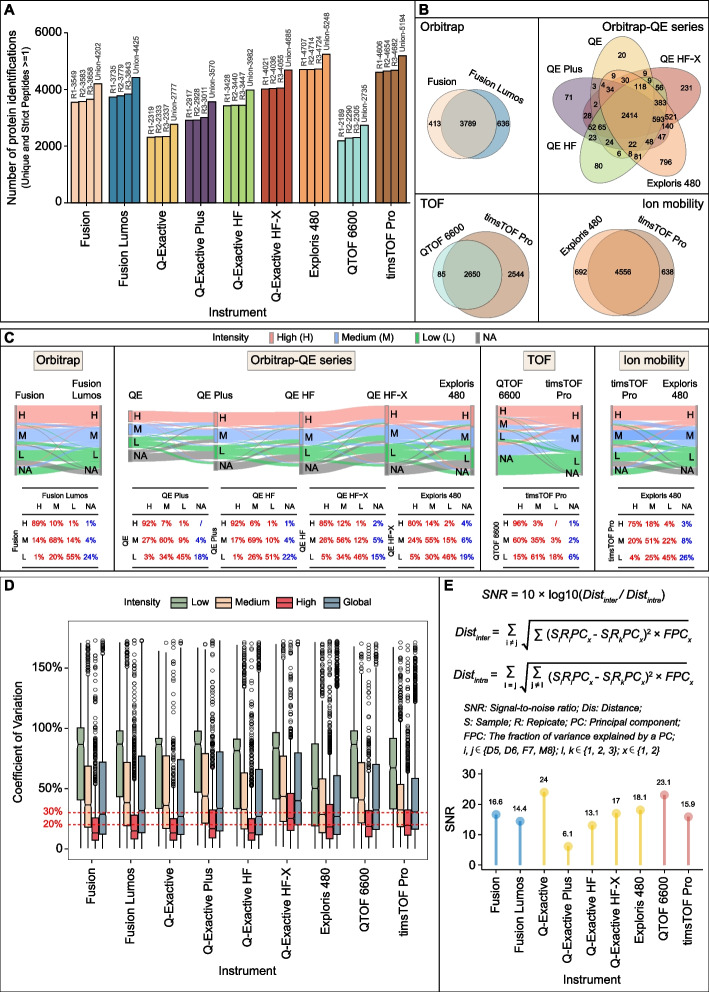
Variation among nine conventional instruments. **A** Number of proteins identified in sample D5 (one of the twin daughters) from the Quartet using nine conventional instruments. **B** Venn diagram of proteins quantified from the same series of MS instruments, corresponding to Orbitrap Fusion series, Orbitrap QE series, TOF series, and ion mobility series. **C** Sankey plot (top panel) depicting the flow direction of reproducibility for proteins within different (low, medium, high) intensity groups along with upgraded instruments. The corresponding and detailed percentages are summarized in the bottom table. This assessment involved the same four series of MS instruments as described for **B**. For a specific experiment, low intensity refers to the < 33.33th percentile, medium intensity refers to the 33.33–66.7th percentile, and high intensity refers to the > 66.67th percentile. **D** Coefficients of variation (CVs) for proteins within different intensity groups and the global group for the nine conventional instruments (also see Fig. S2B). **E** Definition of signal-to-noise ratio (SNR, top panel) and the SNR distribution of the nine conventional instruments (bottom panel)

Performance comparison of different instruments can provide guidance for optimizing experimental strategies for users while highlighting technological upgrades for manufacturers. We thus analyzed 108 MS files (9 LC–MS/MS × 4 samples × 3 repeats) produced by nine conventional instruments by detecting the Quartet standards in terms of qualitative and quantitative aspects. These instruments were mainly categorized into Orbitrap-type (Fusion series, Fusion and Fusion Lumos; QE series, Q-Exactive, Q-Exactive Plus, Q-Exactive HF, Q-Exactive HF-X, and Exploris 480 with ion mobility) and TOF-type (TripleTOF 6600 and timsTOF Pro with ion mobility) mass spectrometers. We performed four comparisons among the following instruments: (1) two Fusion series instruments, (2) five QE series instruments, (3) two TOF-type instruments, and (4) two instruments equipped with ion mobility. Taking sample D5 as an example, Fusion Lumos identified 4425 proteins, which was 200 proteins more than those identified with Fusion. In the QE series, the five instruments possess upgraded scanning speeds and other configurations introduced with each generation, and the number of proteins identified also gradually increased in parallel with the model upgrade. For example, 2777 proteins were detected using the first generation of QE (Q-Exactive) while 5248 proteins were detected with the latest generation (Exploris 480). For the TOF series, TripleTOF 6600 identified 2735 proteins, whereas timsTOF Pro identified 5194 proteins. Thus, timsTOF Pro and Exploris 480, which are both equipped with ion mobility capability, resulted in comparable protein identification (5194 and 5248, respectively), indicating the advantages of ion mobility in proteome screening (Fig. 4A).

Furthermore, we delineated the protein identification reproducibility of the Fusion, QE, and TOF series and mass spectrometers with embedded ion mobility. As shown in the Venn diagram in Fig. 4B, 90% (3789) of the proteins identified by Fusion were also identified by Fusion Lumos; Exploris 480, as the most advanced instrument in the QE series, covered more than 87% (4452) of the proteins identified by the previous four generations of instruments; timsTOF Pro identified 97% of the proteins detected by TripleTOF 6600 with an additional 2544 specific proteins; and 87% of the proteins identified were common between the two ion mobility-equipped instruments (Exploris 480 and timsTOF Pro). In addition, Exploris and timsTOF pro detected ~ 13% specific proteins (692 and 638 proteins, respectively), suggesting the complementarity of different MS instruments for deep proteome coverage.

The Sankey plot (Fig. 4C) showed that proteins identified by newer-generation instruments covered 90% of those identified by earlier generation instruments. Specifically, in the QE series, 96–100%, 94–96%, and 78–85% of proteins (sum of the red values in each row of the embedded table) in the high-intensity (> 66.67%), medium-intensity (33.33–66.7%), and low-intensity (< 33.33%) expression groups determined by the earlier generation instruments, respectively, were distributed in different intensity groups produced by the newer-generation instruments. The vast majority of proteins in the three different intensity groups were reproducible in the corresponding groups with the same intensity level (Fig. 4C, values on the diagonal in the table) measured by newer-generation instruments, especially those in the high-intensity group. Similar patterns were observed in the comparisons of the Fusion series, TOF-type instruments, and mass spectrometers with ion mobility. This assessment demonstrated that with continuous upgrading of instruments, the qualitative reproducibility of proteins with medium and high intensity is significantly increased (Additional file 1: Fig. S2A) and the detection of low-intensity proteins is improved, which would have a substantial effect on the reproducibility of proteome identification.

The median coefficient of variation (CV) of proteins in the global group of label-free quantitative proteomics generated by different instruments was approximately 30% (Fig. 4D and Additional file 1: Fig. S2B). Q Exactive, Q Exactive HF, and Exploris 480 showed a relatively low median CV (26%) in the global protein group, whereas Q Exactive HF-X showed a higher median CV (40%). Additionally, the median CV of proteins in the high-intensity group was lower than 20%, whereas proteins in the low-intensity group showed high variation, with the median CV value reaching up to 80%.

To objectively evaluate the performance of the MS platforms, we comprehensively considered the inherent differences of the Quartet standards and defined the SNR for quantitatively measuring the overall variations in the instrument or platform based on the inter- and intra-sample distances within principal component analysis dimensions (Fig. 4E, top panel). A higher value indicates that the inherent biological differences between samples have a greater contribution to the total variation relative to that caused by the state of the system or operation procedure. Thus, the SNR score can reflect the performance of an MS platform with respect to the reproducibility of the same sample and the capability of distinguishing different samples. The SNR scores of older versions of the instruments, such as Q-Exactive and TripleTOF 6600 that had lower protein identification coverage, were relatively higher (24 and 23.1, respectively) than those of newer generation versions such as the Fusion series, Q Exactive HF, and timsTOF (Fig. 4E, bottom panel). The proteins identified by earlier generations of mass spectrometers had relatively medium or high abundance, which increased the SNR. Although the newer-generation spectrometers detected more proteins, especially low-abundance proteins, this resulted in more variation in biological quantification differences because of the increase in background complexity, thereby decreasing the SNR.

### Variation among laboratories

**Fig. 5:**
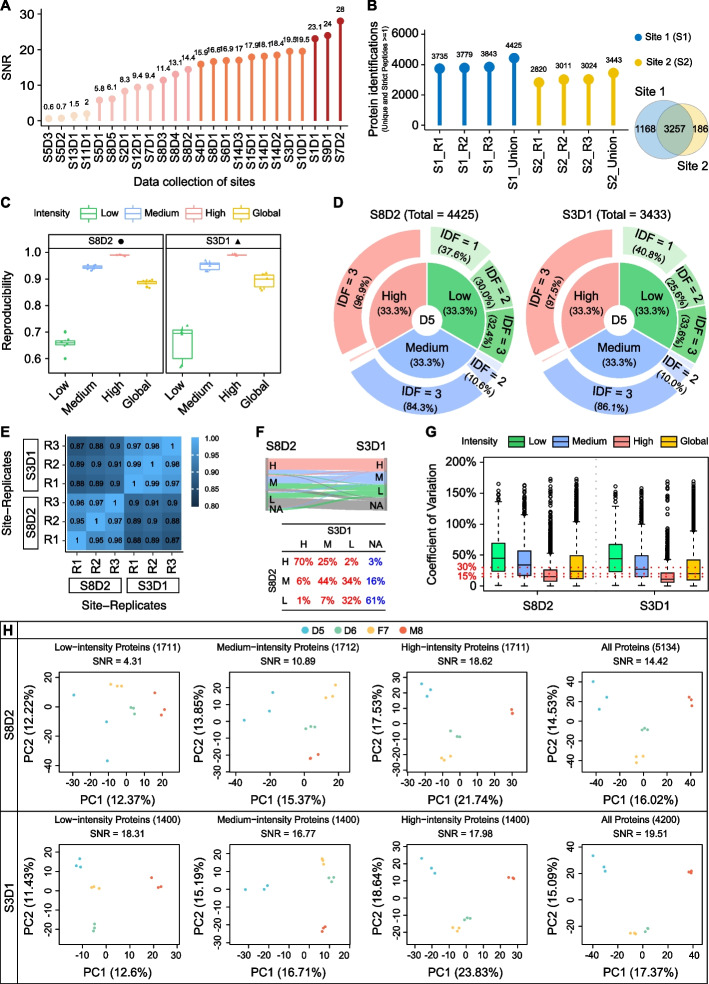
Variation among laboratories. **A** SNR distribution of 24 datasets produced by the nine types of mass spectrometers in 15 laboratories. S: site; D: dataset. **B** Number of proteins identified in S8D2 and S3D1 (left panel) and the Venn diagram of proteins identified from the two datasets (right panel). **C** Reproducibility of detected proteins in low-intensity, medium-intensity, high-intensity, and global groups from the two datasets (left panel: S8D2; right panel: S3D1). **D** Pie chart presenting the percentage of proteins with different identification frequency (IDF) in a specific experiment with three repeats; these proteins are divided into low-intensity, medium-intensity, and high-intensity groups (left panel: S8D2; right panel: S3D1). **E** Pearson’s correlation coefficients matrix representing the correlations between different replicates from two laboratories. **F** Sankey plot (top panel) depicting the flow direction of reproducibility for proteins within different (low, medium, high) intensity groups from S8D2 to S3D1. The corresponding and detailed percentages are summarized in the bottom table. **G** CVs for proteins within different intensity groups and the global group of S8D2 and S3D1. **H** Principal component analysis and SNR scoring for S8D2 and S3D1 using proteins within different intensity groups and the global group

Quantitative evaluation based on the SNR, which ranged from 0.6 to 28, demonstrated substantial variation in the proteomic detection level of different platforms (Fig. 5A). An SNR of < 1 indicates that the variation introduced by system performance or operation procedure obscured inter-sample biological differences. Here, we judged results with an SNR of 0–2 as “ineligible,” 2–10 as “average,” 10–20 as “good,” and > 20 as “excellent,” which accounted for 16.7, 20.8, 50, and 12.5% of all 24 experiments, respectively. These results indicate that biological differences identified by most proteomics platforms (> 80%) were reliable and highlight the necessity of well-designed reference materials and suitable metrics in performance evaluation among different platforms.

We next selected 2 of the 20 qualified experiments for qualitative and quantitative inter-laboratory performance comparison. Two laboratories, Site 3 and Site 8, generated MS datasets S3D1 and S8D2, respectively, using Fusion Lumos. Taking sample D5 as an example, S8D2 included 4425 proteins, which was 982 proteins more than those identified in S3D1 (Fig. 5B, left panel). Overall, 94.6% (3257) of the 3443 proteins in S3D1 were included in S8D2, with 1168 and 186 site-specific proteins in S8D2 and S3D1 (Fig. 5B, right panel), respectively, demonstrating that the proteomics operation procedure at Site 8 was more suitable for in-depth proteome analysis. We then divided the proteins in S3D1 and S8D2 into low-, medium-, and high-intensity groups. Compared with S3D1, in-depth protein identification in S8D2 greatly improved protein reproducibility, especially in the low- and medium-intensity groups with a narrow interquartile range (Fig. 5C). The corresponding pie chart further strengthened this conclusion. For example, in the low-intensity group, 3.2% more proteins had an identification frequency > 1 in S8D2 than in S3D1 (62.4% vs. 59.2%; Fig. 5D). Pearson’s correlation coefficient between samples in S3D1 ranged from 0.97 to 0.99, which was superior to that of 0.95–0.97 in S8D2 (Fig. 5E). The Sankey plot indicated that proteins identified in S3D1 only covered ~ 73.6% (Fig. 5F, total proportion of the sum of the red values in each column of the table) of those identified in S8D2. Specifically, 97, 84, and 40% of proteins (Fig. 5F, the sum of the red values in each row of the table) in the high-, medium-, and low-intensity groups determined in S8D2 were distributed in different intensity groups than those in S3D1. These results indicate that compared with Site 3, Site 8 has advantages in detecting low-intensity proteins. The median CVs in the global group for label-free quantification in S3D1 and S8D2 were 20 and 24% (Fig. 5G and Additional file 1: Fig. S3 A), respectively, indicating good quantification consistency.

Nevertheless, Site 3 showed a significant advantage over Site 8 in measuring the built-in difference of the Quartet standards with respect to the SNR (19.51 vs. 14.42) (Additional file 1: Fig. S3 B); the SNRs gradually increased according to the increase in protein abundance (Fig. 5H). The SNR of the high-intensity sub-proteomes was comparable between S3D1 and S8D2, and although the SNR of the low- and medium-intensity sub-proteomes dramatically decreased to 4.31 and 10.89 in S8D2, it remained high for S3D1 (18.31 and 16.77, respectively). Therefore, we deduced that SNR scores were significantly more affected by the low- and medium-intensity sub-proteomes than by the high-intensity sub-proteome.

Collectively, these results demonstrate notable inter-laboratory differences in both qualitative and quantitative aspects, although most laboratories were able to identify biological differences; thus, it is necessary to perform inter-comparisons using the reference material. Such inter-laboratory comparisons can help laboratories validate the reliability of an SOP by determining the standard deviations of reproducibility and SNR and ultimately produce more confident results.

### Long-term stability of standards

**Fig. 6:**
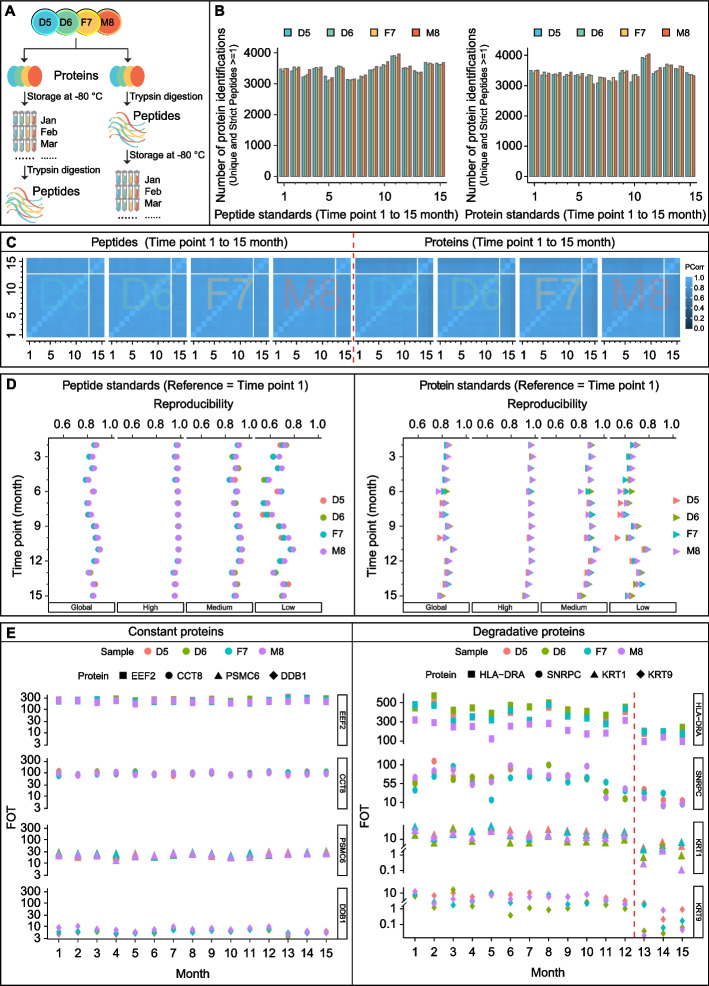
Long-term stability of standards. **A** Longitudinal study design of stability evaluation of the Quartet standards. **B** Number of proteins identified monthly at 15 different time points. Left panel: peptide standards; Right panel: protein standards. **C** Pearson’s correlation coefficients matrix representing the correlation coefficients between the different mass spectrometry runs generated across 15 months using the Quartet standards (left panel: peptide standards; right panel: protein standards). **D** Reproducibility of proteins within different intensity groups and the global group during 15-month monitoring (left panel: peptide standards; right panel: protein standards). **E** Quantitative expression patterns of some proteins over 15 months. Left panel: proteins with high stability over 15 months; right panel: proteins with a degradation trend, especially after 12 months

To evaluate the stability of the Quartet standards, we conducted 15-month longitudinal monitoring for both peptides and proteins. All Quartet standards were stored at − 80 °C until analysis. Standards in protein form were tryptic-digested into peptides and then subjected to LC–MS/MS analysis, whereas standards in peptide form were directly loaded onto the LC–MS/MS system after dissolution. All stability evaluations were performed on the same Fusion Lumos instrument using the single-shot strategy. The protein and peptide form standards were tested monthly, generating a total of 120 MS files (2 types of standards × 4 samples × 15 months) (Fig. 6A).

Little qualitative difference was found between the Quartet standards in peptide and protein forms with respect to the number of proteins identified at each monitoring point, with an average of 3447 proteins identified at the 15 different time points (Fig. 6B). From a quantitative perspective, the proteomes had high correlations among experiments conducted in the first 12 months but were significantly weaker for the experiments performed in the last 3 months (Fig. 6C). This difference may have resulted from the degradation of peptides or proteins in the Quartet standards over time. Furthermore, we evaluated the reproducibility of proteins identified in the Quartet standards in peptide and protein forms, using the proteins identified in the first month as the reference (Fig. 6D). For standards in peptide form, the reproducibility of identified proteins showed high consistency, although there was a slight fluctuation trend, which was mainly caused by proteins in the low-intensity group. In the medium- and high-intensity protein groups, the inflection point of reproducibility occurred in the 13th month, which may have been due to the degradation of a few peptides. Comparatively, for standards in protein form, protein reproducibility started to show a relatively sharp decline by the 12th month for all three intensity groups. We speculated that this was also likely due to the degradation of more proteins. The stability of the prepared Quartet standards, in both peptide and protein forms, was high and similar for 1 year. Thereafter, the stability of standards in peptide form was better than in protein form. With respect to the quantitative expression patterns, some proteins in either medium- or high-intensity group, such as EEF2, CCT8, PSMC6, and DDB1, maintained high stability over 15 months, indicating their potential as internal standards (Fig. 6E, left panel). In addition, some proteins distributed in different intensity groups, such as HLA − DRA, SNRPC, KRT1, and KRT9, degraded over time, especially after 12 months (Fig. 6E, right panel). Taken together, these results demonstrate that both protein and peptide samples can be stably stored for 1 year at − 80 °C, with degradation occurring thereafter, and that the peptide form is more stable and reliable during storage than the protein form.

### Injection order contributes to proteome differences

**Fig. 7:**
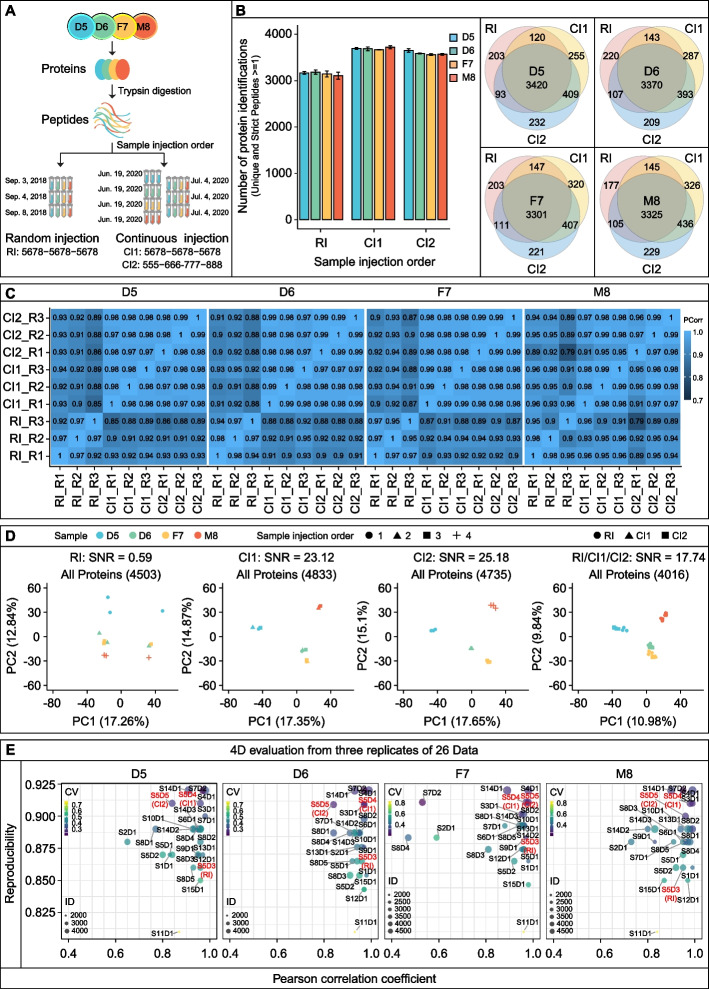
Injection order contributes to proteome differences. **A** Experimental study design. **B** Proteins identified in the Quartet samples using three different injection orders (left) and Venn diagram of the identified proteins (right). **C** Pearson’s correlation coefficients matrix representing the correlation coefficients between different mass spectrometry runs generated by three different injection orders. **D** Principal component analysis and signal-to-noise ratio (SNR) scores for datasets produced using different injection orders and the integrated dataset. **E** Four-dimensional visualization map. RI: random injection order (5678–5678-5678); CI1: continuous injection order 1 (5678–5678-5678); CI2: continuous injection order 2 (555–666-777–888)

We next evaluated the influence of the injection order on the quantitative capability of the MS systems. To this end, we compared the following three modes of injection order: random injective mode (RI), in which the Quartet standards were randomly injected into Q-Exactive HFX at any point within 1 week; continuous injection 1 (CI1), in which the Quartet standards were injected continuously into the same instrument (Q-Exactive HFX) in the order 5678–5678-5678; and continuous injection 2 (CI2), in which the Quartet standards were injected continuously into the same instrument (Q-Exactive HFX) in the order 555–666-777–888 (Fig. 7A). The proteins identified via the three modes showed more than 80% overlap, demonstrating that the injection order did not substantially affect the qualitative performance of the MS system (Fig. 7B). As shown in Fig. 7C, CI2 mode showed the highest quantification correlation among the three modes (Pearson’s correlation coefficient: 0.96–0.99), whereas the RI mode had the lowest quantitative performance (Pearson’s correlation coefficient: 0.90–0.98).

We also used the SNR indicator to evaluate the ability of the three injection modes to detect differences between the Quartet standard samples. The SNR of datasets produced in RI mode was only 0.59, indicating that the batch effect introduced by random injection had concealed biological differences. In contrast, the continuous injection strategy resulted in an excellent SNR score of 23.12 for CI1 mode and 25.18 for CI2 mode (Fig. 7D). Integration analysis of the data from the three injection modes showed an overall SNR of 17.74, suggesting that multiple repeats of samples reduce the batch effect. We thus proposed a four-dimensional visualization map (Fig. 7E) comprising protein identification, protein reproducibility, correlation among experiments, and experimental CV, which can be used to measure the proteomics operation level of each laboratory in a comprehensive manner, note the advantages and disadvantages of each laboratory, and highlight directions for further improvement.

### Absolute quantification of Quartet standards

To promote the large-scale, worldwide application of the Quartet as a proteome reference material, it is necessary to calculate the absolute number of the whole proteome (copy number per cell) according to the international metrological rules established by the International Organization for Standardization (ISO). To this end, we used the QconCAT method to measure the internal standard “anchor” proteins. We randomly selected 33 proteins with intensity-based absolute quantification (iBAQ) values distributed in four orders of magnitude as anchor proteins for absolute quantification and calibration of the copy numbers for the whole proteome. Representative unique peptides from these 33 anchor proteins were designed as QconCAT proteins by tandem fusion and split into 9 QconCAT proteins. Over 99% of the QconCAT proteins grown in the heavy isotope medium incorporated the labeled lysine, demonstrating the effectiveness of this approach.

**Fig. 8:**
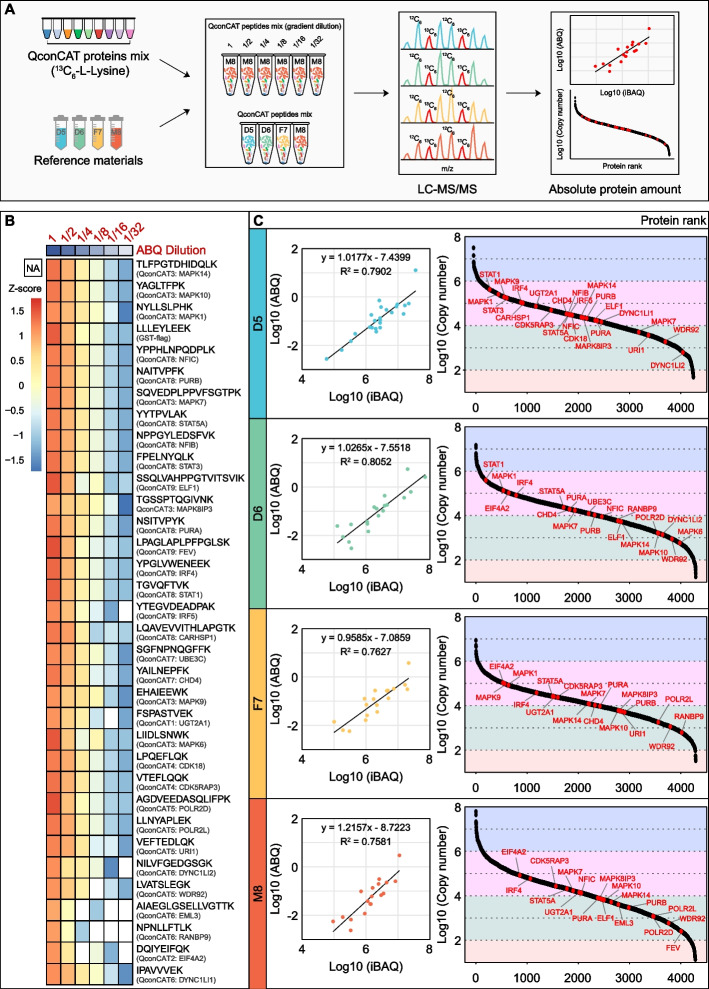
Absolute quantification of Quartet standards. **A** Workflows for absolute quantification (ABQ) of the Quartet. **B** Heatmap showing the linearity of peptide candidates as internal standards under a dilution series. **C** Response curves between ABQs and iBAQs of the anchor proteins (internal standard) in each sample of the Quartet (left), and dynamic ranges of ABQs (copy number/cell) for each sample of the Quartet (right)

To determine the absolute molar value of the C^13^-labeled QconCAT proteins, we synthesized the gold C^12^ GST peptide LLLEYLEEK (Institute of Metrology) and added gold peptides to nine tubes corresponding to the nine QconCAT proteins, which were digested with trypsin and loaded into the LC–MS/MS system for detection (Additional file 1: Fig. S4 A). The molar amount of each QconCAT protein can then be calculated (Additional file 5: Table S4). For Quartet standard quantification, a dilution series of the C^13^-labeled QconCAT peptides was mixed with the Quartet peptide standard samples and then subjected to LC–MS/MS analysis (Fig. 8A). The corresponding heatmap portrays the linear trend of all identified C^13^-labeled anchor peptides (Fig. 8B and Additional file 6: Table S5). A dilution response curve of every C^13^-labeled anchor peptide (including GST peptide LLLEYLEEK) in the QconCAT proteins showed excellent linearity (Additional file 1: Fig. S4 B-D), suggesting the good quantification accuracy of QconCAT proteins and the reliability of endogenous proteins in the Quartet anchored by QconCAT proteins.

The absolute molar value of the corresponding proteins in the Quartet standard sample was determined based on quantification of the C^13^-labeled anchor peptides (Additional file 7: Table S6). The copy number of the 33 anchor proteins ranged from 10^2^ to 10^6^ copies/cell. We aligned the absolute copy numbers (ABQ) of the anchor proteins to their corresponding iBAQ values and found that the ABQs and iBAQs were well-correlated (R^2^ = 0.7581–0.8052) (Fig. 8C), demonstrating consistency between relative quantification (iBAQ) and absolute quantification (copy number). Finally, we quantified the abundance of more than 4000 proteins in each sample of the Quartet reference materials by aligning the proteome to the anchor proteins (Additional file 7: Table S6). Their dynamic ranges spanned over ~ 7 orders of magnitude from 10^1^ to 10^8^ copies/cell.

The parallel reaction monitoring (PRM) assays are powerful targeted approaches to detect and quantify pre-specified proteins with a high throughput using high-resolution mass spectrometers. To further validate the “anchor” proteins for potential calibration, we employed the targeted MS approach, PRM assays, to perform targeted assays on the Quartet samples (D5, D6, F7, and M8, × 3 repeats). Using search results from previous data, we directly selected a set of target peptides (Additional file 8: Table S7) that are unique to “anchor” proteins (CARHSP1, CDK5RAP3, CHD4, DYNC1LI1, EIF4A2, ELF1, EML3, IRF4, IRF5, MAPK1, MAPK14, POLR2D, PURA, STAT1, STAT3) and designed the parallel reaction monitoring (PRM) method [34]. Based on PRM quantification, we observed that these signature proteins can be detected in the Quartet samples, and the quantitative results show consistency within the same samples and heterogeneity between different samples (Additional file 1: Fig. S5). In conclusion, our data demonstrated the stable expression of the “anchor” proteins were validated by PRM approach in the Quartet samples. Thus, the absolute quantification of the Quartet proteome potential calibration with “anchor” proteins have certain reliability, which also can provide reference datasets for QC and quality assessment of an LC–MS/MS platform for both basic research and clinical applications.

## Discussion

Quantitative proteomics is playing an increasingly prominent role in clinical and basic research and has been gradually introduced into clinical practice. Proteomic molecular subtyping research led by the Clinical Proteomic Tumor Analysis Consortium (CPTAC) and the Chinese Human Proteome Project (CNHPP) consortia dissected the proteomes of several cancer types, including brain cancer [35], diffuse gliomas [36], pituitary neuroendocrine tumor [37], hepatocellular carcinoma [38, 39], lung cancer [40], lung adenocarcinoma [41, 42], gastric cancer [43], colon cancer [44–46], clear cell renal cell carcinoma [47], endometrial carcinoma [48], ovarian cancer [49], prostate cancer [50], and breast cancer [51, 52]. These projects proposed druggable targets and indicators for selecting clinical treatment strategies. Moreover, proteomics analyses of blood, urine, and cerebrospinal fluid samples have facilitated the discovery of several molecular markers of human diseases [53–55].

To guarantee the reproducibility of the molecular subtyping and markers revealed by proteomics, it is vital to conduct a thorough and objective multi-platform assessment of the utility of particular proteomics technologies. These evaluations require reference materials as benchmarks. Additionally, in the above CPTAC and CNHPP projects, multi-omics strategies have also become a routine approach. In these studies, a sample is usually divided into three (or four) parts so that DNA, RNA, and protein (or metabolites) can be extracted simultaneously during the sample preparation process, and then used for multi-omics data generation and integrated analysis. Therefore, multi-omics reference materials and relevant QC metrics from the same sample of interconnected reference materials are required for quality assessment of each omics measurement. In this context, we developed the Quartet reference material and carried out the evaluation work. Our work has the following five distinct characteristics:

- Quartet family-based multi-omics study on the same sample.
  We launched the Quartet Project to provide multi-omics “ground truth” as well as best practices for the QC and data integration of multi-omics profiling. The Quartet multi-omics reference material suites included DNA, RNA, proteins, and metabolites derived from a quartet family of parents (F7 and M8) and monozygotic twin daughters (D5 and D6). Two types of “built-in truth” are provided for quality assessment of multi-omics profiling. One is the ability to correctly distinguish samples into four clusters (D5, D6, F7, and M8). For each omics type, their data generation and analysis must have the basic ability to differentiate the four different biological samples from technical replicates. The other one is the ability to correctly identify the hierarchical relationships across multi-omics features according to the rule of the central dogma, which can be used for assessing the reliability of correlation-based multi-omics network integration (Additional file 1: Fig. S6).
- Quartet family-based inter- and intra-group different analysis through “SNR”.
  In our study, proteomic difference (built-in truth) from four individuals can be clearly depicted in two-dimensional (2-D) PCA plot. Four samples could be completely separated by the use of PCA, three replicates of each sample clustered compactly. Based on the Quartet sample design and their visualization in 2-D PCA plot, we can calculate the intrinsic biological differences (“signal”) among the Quartet samples and variations among technical replicates within the same sample group (“noise”). This allows us to define the “SNR” metric as the ratio between “signal” and “noise,” which can gauge the performance of a platform, a lab, a protocol, or a batch.
- Providing finely differentially expressed proteins (DEPs) with confidence interval.
  Based on the Quartet sample design, the comparative proteomics performance of distinguishing built-in truth from different biological samples can be easily evaluated for multiple instruments or at multiple labs. We counted the occurrence frequency of up- and downregulated DEPs in the 24 experiments by pairwise comparisons between samples within each experiment, and divided them into eight intervals according to the reproducibility frequency of DEPs. These proteins can be used as a benchmark dataset to evaluate the quantitative ability of comparative proteomics on different platforms.
- Absolute quantification of the Quartet protein reference materials.
  To promote the large-scale, worldwide application of the Quartet as a proteome reference material, it is necessary to calculate the absolute number of the whole proteome (copy number per cell) according to the international metrological rules established by the International Organization for Standardization (ISO). To this end, we used the QconCAT (Methods Enzymol, 2015, PMID: 26,791,984) method to measure the internal standard “anchor” proteins, and conducted PRM validation analysis for some “anchor” proteins. Absolute quantification of the Quartet proteome can provide reference datasets for QC and quality assessment of an LC–MS/MS platform for both basic research and clinical applications.
- Proteome reference material establishment complying with the First Class of National Reference Materials will serve the proteomics community.
  The Quartet Project provides the community with multi-omics (Genomic, transcriptomic, proteomic and Metabolomic) reference materials and reference datasets of four samples from a family Quartet, which are 1:1 matched, for objectively assessing quality in data generation and integrated analysis in increasingly large-scale multi-omics studies. It is worth mentioning that the DNA and RNA reference material suites have been approved by China’s State Administration for Market Regulation as the First Class of National Reference Materials (Standard Material Numbers: GBW0900-GBW0907) and are extensively being utilized for proficiency testing and method validation. As a part of the Quartet Project for the quality control and data integration of multi-omics profiling, this Quartet proteomic protein standard reference materials are also in the national first-class reference materials approval process. After formal approval and release, the standard reference materials can not only be shared with various platforms, but also obtain standard reference datasets of materials through the “National Metrological Science Data Center” (https://srd.nmdc.ac.cn/gene/chinese-quartet/) for quality assessment between laboratories.

Furthermore, it would be valuable to discuss the impact and significance of the chromatographic system when comparing and evaluating different MS instrument performances. To assess the effect of different chromatographic instruments on proteome detection, we compared two different systems, the Vanquish Neo and EASY nLC 1200, coupled to the same MS instrument (Orbitrap Ascend Tribrid; Thermo Fisher Scientific, Rockford, IL, USA). We used the Quartet D5 sample and found that the total ion chromatograms (TIC) of the two MS detection results were consistent, with comparable retention times for representative peptides (within 1 min) (Additional file 1: Fig. S7). Strict control of the liquid phase system parameters can minimize the influence of different chromatographic instruments on overall detection performance, enabling comparison of MS instrument performance.

In summary, on the basis of the previous QC studies, we further developed multi-omics reference materials suites, which could be used for simultaneously evaluating between- and within-group differences based on the Quartet sample design containing pedigree information. In our reference datasets, “ground truth” of proteins not only contain detection frequency and average expression but also include their differential expression attributes. Cross-omics DEPs were also annotated. Additionally, for each protein in reference datasets, their relative and absolute quantification are provided. The Quartet protein reference materials are established by complying with the ISO, and have been publicly available at chinese-quartet.org, as well as currently in the process of being approved.

Our work is a crucial component of the MAQC-IV and provides the life science community with a unique and valuable proteome resources, which could serve as a reference for the research community to evaluate new technologies, labs, assays, products, lab operators, and computational algorithms. Additionally, this work not only advances the field of proteomic quality control research based on previous work, but also contributes the proteomics power to conducting high-quality, large-scale multi-omics studies. We strongly recommend the use of Quartet reference materials to optimize experimental methods and monitor the status of experimental platforms for producing high-quality data, and discovering more useful knowledge, thereby promoting the development of precision medicine.

## Conclusions

In summary, we established proteome standard reference materials, termed the Quartet, with absolute protein expression reference values. A total of 816 MS files were thoroughly profiled, and the qualitative and quantitative characteristics of the Quartet standard samples were determined, providing valuable reference datasets for proteomics analyses. As the Quartet standards were developed within the MAQC-IV project, the DNA, RNA, and metabolite forms from the same batch of samples are also available, thereby allowing for trans-omics integration of the corresponding genome, transcriptome, and metabolome datasets. We believe that the Quartet protein standards, along with other biomaterial forms, will have broad applications in basic research and translational medicine.

## Methods

### Initiation and progression of the Quartet project

This study was initiated by Fudan University in collaboration with over 30 institutions worldwide under the supervision and guidance of the National Institute of Metrology. As part of the Taizhou Longitudinal Study in Taizhou, Jiangsu, China, four healthy volunteers from a family Quartet, including father (F7), mother (M8), and monozygotic twin daughters (D5 and D6), were recruited and their peripheral blood was collected to establish the human immortalized cell lines. Large quantities of multi-omics reference materials suites (DNA, RNA, protein, and metabolite) were established simultaneously from these four immortalized cell lines. Each reference material was stored in over 1000 vials. They are applicable to a wide range of multi-omics technologies, including DNA sequencing, DNA methylation, RNAseq, miRNAseq, LC–MS/MS-based proteomics, and metabolomics. This study was conducted in accordance with the principles of the Declaration of Helsinki and was approved by the Ethics Committee of the School of Life Sciences, Fudan University (BE2050). All four donors signed informed consent forms. As a part of the Quartet Project for the quality control and data integration of multi-omics profiling, the study of proteomic protein standard reference materials and datasets were uniformly deployed by Fudan University in 15 laboratories from six cities in China.

### Establishment of the Quartet cell lines

For establishing cell lines of genome, transcriptome, proteome, and metabolome reference materials, we adopted the same widely used protocol in the same laboratory of using Epstein-Barr virus (EBV) to establish immortalized lymphoblastoid cell lines (LCLs) [56]. Peripheral blood mononuclear cells (PBMCs) were isolated by Ficoll Lymphocyte Separation Solution. Naïve B cells were enriched by EasySep Human naïve B Cell Enrichment Kit (STEMCELL Technologies) and infected with Epstein-Barr virus (EBV) by centrifugation at 2000 rpm for 1 h. After incubation, the successfully infected and immortalized cells were propagated in culture medium.

### Cell culture and collection

The Quartet cell lines were cultured in RPMI 1640 with 2 mM L-glutamine, 10% heat-inactivated FBS (fetal bovine serum), 1% penicillin, and 1% streptomycin (all from Sigma-Aldrich, Gillingham, UK) at 37 °C with 5% CO_2_. The cells were passaged every 72 h at a 1:4 split ratio. To obtain the multi-omics reference materials (DNA, RNA, protein, and metabolite), 2 × 10^9^ cells were cultured and harvested simultaneously for each type of cell line in each omics. Specifically, the cells grew in suspension and were centrifuged at 300* g* for 5 min to obtain cell pellets. Mixed 2 × 10^9^ cells from the same type of cell line, collected them, and washed them twice with cold PBS. The cell pellets were stored at − 80 °C until used as reference materials for the preparation of DNA, RNA, protein, and metabolite.

### Preparation of the protein reference materials

To obtain the protein reference materials, 2 × 10^9^ cells were collected from each cell line of each type, lysed in lysis buffer (8 M urea, 100 mM ammonium bicarbonate, pH 8.0) supplemented with protease inhibitors for 10 min on ice, and then sonicated for 3 min (2 s on and 2 s off) on ice and centrifuged at 16,000 × *g* for 20 min to remove the cell debris. The supernatants were collected, and the protein concentration was measured using a bicinchoninic acid protein assay. Extracted proteins were loaded into 10-kD Microcon filtration devices (Millipore, Burlington, MA), centrifuged at 16,000 × *g* for 20 min, and washed twice with urea lysis buffer and twice with 50 mM NH_4_HCO_3_. The samples were then digested using trypsin at an enzyme: protein mass ratio of 1:25 overnight at 37 °C, after which peptides were extracted and dried (SpeedVac; Eppendorf, Hamburg, Germany). Two types of reference materials derived from 2 × 10^9^ cells of each of the four family members were uniformly prepared and sub-packed into 1000 EP tubes with specific color labels (D5: blue, D6: green, F7: yellow, and M8: red) and stored in a − 80 °C freezer. For generating the protein reference material, aliquots of 100 µg total protein were stored in each tube. For generating the peptide reference material, aliquots of 10 µg total tryptic peptide were stored in each tube. Before use, the protein reference materials were digested into peptides. We distributed these aliquots to 15 laboratories throughout six cities in China to participate in this testing (Fig. 1a). Subsequently, while applying for the First Class of National Reference Materials, the remaining two types of eight reference materials (four proteins and four peptides) have been uniformly transported to the National Institute of Metrology, providing convenience for laboratories around the world to obtain quality control materials through official channels.

### Preparation of QconCAT proteins

QconCAT proteins were prepared according to the procedures reported by Ding et al. [57]. Briefly, the QconCAT cDNA was reverse-translated from amino acid sequences of the selected QconCAT tryptic peptides and chemically synthesized (Generay Biotech, Shanghai, China) before cloning into the pGEX4T-1 vector (Addgene, Watertown, MA). The GST-QconCAT plasmids were then transformed into *Escherichia coli* BL21 cells (TransGen Biotech, Beijing, China) for protein expression. A fresh *E. coli* BL21 culture was inoculated into 5 mL of heavy SILAC Dulbecco’s modified Eagle’s medium (^13^C_6_ lysine, without glutamine; Invitrogen, Carlsbad, CA) and grown at 37 °C for 16 h. Isopropyl-β-d-1-thiogalactopyranoside (0.4 mM; Sigma-Aldrich, St. Louis, MO) was added to the bacterial culture when the absorbance at 600 nm reached ∼0.5 to induce QconCAT protein expression at 37 °C for 3 h. The expression and identity of the recombinant protein was verified by mass spectrometry. The BL21 cells were collected, suspended in NETN buffer (150 mM NaCl, 1 mM EDTA, 50 mM Tris–HCl, 1% NP-40, with protease inhibitors), and lysed on ice by sonication. The lysate was then centrifuged at 60,000 rpm for 10 min, and the supernatant was collected to purify GST-QconCAT recombinant protein using GSH beads. The purified GST-QconCAT proteins were eluted by elution buffer (10 mM glutathione, 50 mM Tris–HCl, 5% glycerol) and stored at − 80 °C until use.

### Application of standard reference materials

The Quartet Project provides the community with multi-omics (Genomic, transcriptomic, proteomic and Metabolomic) reference materials and reference datasets of four samples from a family Quartet, which are 1:1 matched, for objectively assessing quality in data generation and integrated analysis in increasingly large-scale multi-omics studies. It is worth mentioning that the DNA and RNA reference material suites have been approved by China’s State Administration for Market Regulation as the First Class of National Reference Materials (Standard Material Numbers: GBW0900-GBW0907) and are extensively being utilized for proficiency testing and method validation. As a part of the Quartet Project for the quality control and data integration of multi-omics profiling, this Quartet Human B-lymphocyte proteomic protein standard reference materials are also in the national first-class reference materials approval process. After formal approval and release, the standard reference materials can not only be shared with various platforms, but also obtain standard reference datasets of materials through the “National Metrological Science Data Center” (https://srd.nmdc.ac.cn/gene/chinese-quartet/) for quality assessment between laboratories.

### Protein extraction and tryptic digestion

The cells were lysed in lysis buffer (8 M urea, 100 mM ammonium bicarbonate, pH 8.0) supplemented with protease inhibitors for 10 min on ice, and then sonicated for 3 min (2 s on and 2 s off) on ice and centrifuged at 16,000 × *g* for 20 min to remove the cell debris. The supernatants were collected, and the protein concentration was measured using a bicinchoninic acid protein assay. Extracted proteins were loaded into 10-kD Microcon filtration devices (Millipore, Burlington, MA), centrifuged at 16,000 × *g* for 20 min, and washed twice with urea lysis buffer and twice with 50 mM NH_4_HCO_3_. The samples were then digested using trypsin at an enzyme:protein mass ratio of 1:25 overnight at 37 °C, after which peptides were extracted and dried (SpeedVac; Eppendorf, Hamburg, Germany). Two types of proteome standards in peptide and protein forms were uniformly prepared and sub-packed into EP tubes, followed by storage in a − 80℃ freezer. Before use, the protein standards were digested into peptides.

### One-dimensional reversed-phase separation

The dried peptides were loaded into a home-made Durashell reverse-phase column (2 mg packing, 3 μm, 150 Å, in a 200-μL tip; Agela, Torrance, CA), and then sequentially eluted with different gradients of elution buffer containing mobile phase A [2% acetonitrile (ACN), adjusted to pH 10.0 using NH_3_·H_2_O) and different concentrations of mobile phase B (98% ACN, adjusted to pH 10.0 using NH_3_·H_2_O). The different fractions (6, 12, 18, or 30) were then vacuum dried for sub-sequential MS analysis.

### LC–MS/MS analysis

Samples were analyzed on Orbitrap Fusion, Orbitrap Fusion Lumos, Q-Exactive, Q-Exactive Plus, Q-Exactive HF, Q-Exactive HF-X, and Exploris 480 mass spectrometers (Thermo Fisher Scientific, Rockford, IL) coupled with a high-performance liquid chromatography system (EASY nLC 1000, Thermo Fisher Scientific), or a TripleTOF 6600 + (Sciex, Concord, Ontario) connected to Ultra Plus NanoLC 2D HPLC system (Eksigent Technologies, Dublin, CA), or a timsTOF Pro (Bruker Daltonics) set to acquire data in Parallel Accumulation Serial Fragmentation (PASEF) mode mass spectrometer connected to nanoElute (Bruker Daltonics). Dried peptide samples re-dissolved in Solvent A (0.1% formic acid in water) were loaded onto a 2-cm self-packed trap column (100 μm inner diameter, 3 μm ReproSil-Pur C18-AQ beads, Dr. Maisch GmbH) and separated on a 150-μm-inner-diameter column with a length of 15 cm (1.9 μm ReproSil-Pur C18-AQ beads, Dr. Maisch GmbH) over a 75-min gradient (Solvent A: 0.1% formic acid in water; Solvent B: 0.1% formic acid in 80% ACN) at a constant flow rate of 600 nL/min (0–75 min, 0 min, 4% B; 0–10 min, 4–15% B; 10–60 min, 15–30% B; 60–69 min, 30–50% B; 69–70 min, 50–100% B; 70–75 min, 100% B). (a) MS analysis (QE series). Eluted peptides were ionized at 2 kV and introduced into the mass spectrometer. Mass spectrometry was performed in data-dependent acquisition mode. For the MS1 Spectra full scan, ions with m/z ranging from 300 to 1400 were acquired by an Orbitrap mass analyzer at a high resolution of 120,000. The automatic gain control (AGC) target value was set to 3E + 06. The maximal ion injection time was 80 ms. Top60 precursors were selected for MS2 experiment. The isolation window of selected precursor was 1.6 m/z. Precursor ions were fragmented with higher energy collision dissociation (HCD) with a normalized collision energy of 27%. Fragment ions were analyzed by an Orbitrap mass analyzer with the resolution of 7,500, AGC target at 5E + 04, as well as the maximum ion injection time of MS2 was 20 ms. Peptides that triggered MS/MS scans were dynamically excluded from further MS/MS scans for 12 s. (b) MS analysis (Fusion series). Eluted peptides were ionized at 2 kV and introduced into the mass spectrometer. Mass spectrometry was performed in data-dependent acquisition mode. For the MS1 Spectra full scan, ions with m/z ranging from 300 to 1400 were acquired by an Orbitrap mass analyzer at a high resolution of 120,000. The automatic gain control (AGC) target value was set to 3E + 06. The maximal ion injection time was 80 ms. MS2 spectral acquisition was performed in the ion trap in a rapid speed mode with 1.5 s cycletime. Precursor ions were selected and fragmented with higher energy collision dissociation (HCD) with a normalized collision energy of 27%. Fragment ions were analyzed by an ion trap mass analyzer with an AGC target at 5E + 04. The maximal ion injection time of MS2 was 20 ms. Peptides that triggered MS/MS scans were dynamically excluded from further MS/MS scans for 12 s. The coefficients of variation (CV) values on FAIMS were − 45 V and − 65 V. (c) MS analysis (TOF series). For TripleTOF 6600 + , mass spectrometry was performed in data-dependent acquisition mode. MS1 spectra were collected between 300 and 1200 m/z for 250 ms. The 27 most intense precursor ions with charge states 2–4 that exceeded 300 counts per second were selected for fragmentation, and the corresponding fragmentation MS2 spectra were collected between 100 and 1700 m/z for 100 ms. After the fragmentation event, the precursor ions were dynamically excluded from reselection for 12 s. The mass spectrometer was a timsTOF Pro (Bruker Daltonics) set to acquire data in Parallel Accumulation Serial Fragmentation (PASEF) mode. The TIMS accumulation time was set to 100 ms and precursor masses for 0.4 min where charge states of 2–4 were allowed. The resolution parameter was set to 50,000 for MS1 and MS2. Mass spectra for MS1 and MS2 scans were recorded between 100 and 1700 m/z. Ion mobility resolution was set to 0.60–1.60 V·s/cm over a ramp time of 100 ms. Data-dependent acquisition (DDA) was performed using 10 PASEF MS/MS scans per cycle with a near 100% duty cycle. An active exclusion time of 0.4 min was applied to precursors that reached 20,000 intensity units.

### Peptide and protein identification

MS raw files were processed using the “Firmiana” [33] proteomics workstation. Briefly, raw files were searched against the NCBI human Refseq protein database using the Mascot search engine (Matrix Science Inc). The mass tolerances were as follows: 20 ppm for precursor and 0.5 Da for product ions collected by Fusion series, 20 ppm for precursor and 50 mmu for product ions collected by QE series, 15 ppm for precursor and 0.05 Da for product ions collected by TOF series. Up to two missed cleavages were allowed. The database searching considered cysteine carbamidomethylation as a fixed modification, and N-acetylation, and oxidation of methionine as variable modifications. Precursor ion score charges were limited to + 2, + 3, and + 4. For the quality control of protein identification, the target-decoy-based strategy was applied to confirm the FDR of both peptide and protein, which was lower than 1%. Percolator was used to obtain the quality value (*q*-value), validating the FDR (measured by the decoy hits) of every peptide-spectrum match (PSM), which was lower than 1%. Subsequently, all the peptides shorter than seven amino acids were removed. The cutoff ion score for peptide identification was 20. All the PSMs in all fractions were combined to comply with a stringent protein quality control strategy. We employed the parsimony principle and dynamically increased the *q*-values of both target and decoy peptide sequences until the corresponding protein FDR was < 1%. Finally, to reduce the false positive rate, the proteins with at least one unique peptide were selected for further investigation [34, 58].

### Label-free-based MS quantification of proteins

The one-stop proteomic cloud platform “Firmiana” [33] was further employed for protein quantification. Identification results and the raw data from the mzXML file were loaded. Then for each identified peptide, the extracted-ion chromatogram (XIC) was extracted by searching against the MS1 based on its identification information, and the abundance was estimated by calculating the area under the extracted XIC curve. For protein abundance calculation, the nonredundant peptide list was used to assemble proteins following the parsimony principle. The protein abundance was estimated using a traditional label-free, intensity-based absolute quantification (iBAQ) algorithm [59], which divided the protein abundance (derived from identified peptides’ intensities) by the number of theoretically observable peptides. Subsequently, the fraction of total (FOT), a relative quantification value was defined as a protein’s iBAQ divided by the total iBAQ of all identified proteins in one experiment, and was calculated as the normalized abundance of a particular protein among experiments. Finally, the FOT values were further multiplied by 10^5^ for ease of presentation, and missing values were assigned 10^−5^ [34, 58].

### Differential protein analysis

The fold-change in expression level was used to determine whether proteins were differentially expressed between samples. Proteins with expression level changes greater than twofold were considered to be upregulated or downregulated. The proteins identified from MS runs at the participating laboratory for each sample in the Quartet were divided into multiple groups according to the confidence intervals of their occurrence frequency in all experiments (observation times/total measurement times).

### The SNR calculation

Signal-to-noise ratio (SNR) is a measurement used in science and engineering. SNR is defined as the ratio of the power of a signal to the power of noise and is often expressed in decibels (https://en.wikipedia.org/wiki/Signal-to-noise_ratio). In this study, the average distances representing the intrinsic “differences” among distinct biological sample groups are regarded as the signal, whereas the average distances among technical replicates of the same sample group are regarded as noise. To identify an effective way to characterize the SNR values, we evaluated the performances of SNR values calculated by different algorithms, including Euclidean distance (Dist), overall expression profiles (Expr), Pearson correlation coefficient (Cor), t-Distributed Stochastic Neighbor Embedding (tSNE), and principal components analysis (PCA), and found PCA based SNR outperformed. The numbers of PC components used in calculating SNR were then determined. We decided to use the first two components in PCA to calculate SNR values in correspondence with visualization in PCA plots.

Therefore, SNR is defined as Eq.:

$$SNR=10\times {log}_{10}(\frac{m\times \left(\genfrac{}{}{0pt}{}{n}{2}\right)}{\left(\genfrac{}{}{0pt}{}{m}{2}\right)\times n\times n}\times \frac{\sum_{x=1}^{m-1}\sum_{y=x+1}^{m}\sum_{i=1}^{n}\sum_{j=1}^{n}\sum_{p=1}^{2}{W}_{p}(P{C}_{p,i,x}-P{C}_{p,j,y}{)}^{2}}{\sum_{x=1}^{m}\sum_{i=1}^{n}\sum_{j=i+1}^{n}\sum_{p=1}^{2}{W}_{p}(P{C}_{p,i,x}-P{C}_{p,j,x}{)}^{2}})$$

where *m* is the number of sample groups, while *n* is the number of replicates in each sample group. *W*_*p*_ represents the *p*th principal component of variances. *PC*_*p*,,*x*_, *PC*_*p*,*j*,*x*_ and *PC*_*p*,*j*,*y*_ represent the *p*th component values of replicate *i* and replicate *j* in sample group *x* or sample group *y*, respectively.

A standard sample set consisted of 12 tubes with each representing one of the three replicates of the four protein reference materials. Therefore, a typical SNR in the study was the ratio of the average distances between different biological groups (9*12/2 = 54) to the average distances between technical replicates of the same groups (2*3*4/2 = 12). The distribution of intra-batch SNR values from 24 protein datasets was used to identify a threshold of 12 (mean-standard deviation), indicative of high discriminating power.

### Statistical analysis

Pearson’s correlation coefficient (*r*) was calculated using the cor.test function in the R software. The protein overlap rate was used as a measure of reproducibility, and the CV was used as a measure of quantification consistency. For the heatmap, each protein expression value in the global proteomic expression matrix was transformed into a *Z*-score across all samples. For protein-wise clustering, the distance was set as “Euclidean’’ distance and the weight method was set to “complete.” The *Z*-score-transformed matrix was clustered using the R package pheatmap (version 1.0.12). The SNR was calculated from the inter- and intra-sample distances within principal component analysis dimensions (Fig. 4E, upper panel). All other statistical analyses were performed with RGUI version 3.6 and publicly available on GitHub: https://github.com/ecnuzdd/MAQC/tree/v1.0.0 (under the GNU General Public License v3.0).

### Targeted PRM analysis

Using search results from previous data, a set of target peptides that unique to “anchor” proteins (CARHSP1, CDK5RAP3, CHD4, DYNC1LI1, EIF4A2, ELF1, EML3, IRF4, IRF5, MAPK1, MAPK14, POLR2D, PURA, STAT1, STAT3) was selected and parallel reaction monitoring (PRM) method was designed. An equal amount of each sample (D5, D6, F7, and M8, × 3 repeats) was digested as described in the part of profiling preparation. Peptide samples were injected into the Q-Exactive HF-X Hybrid Quadrupole-Orbitrap Mass Spectrometer (Thermo Scientific) operating in PRM mode with quadrupole isolation and HCD fragmentation. The full MS mode was measured at resolution 60,000 with AGC target value of 3E6 and maximum IT of 20 ms, with scanning range of 300 to 1400 m/z. Target ions were submitted to MS/MS in the HCD cell (1.6 m/z isolation width, 27% normalized collision energy). Fifteen PRM events were performed after MS1 scanning, at resolution 30,000 with AGC target value of 2E5 and maximum IT of 100 ms. Separation was achieved on a 150-μm-inner-diameter column with a length of 15 cm (1.9-μm ReproSil-Pur C18-AQ beads, Dr. Maisch GmbH) in an Easy 1200 nLC HPLC system (Thermo Scientific). Solvent A was 0.1 formic acid in MS-grade water and solvent B was 0.1% formic acid, 80% ACN in MS-grade water. Peptides were separated at 600 nL/min across a gradient ranging from 4 to 100% B over 75 min (0–75 min, 0 min, 4% B; 0–10 min, 4–15% B; 10–60 min, 15–30% B; 60–69 min, 30–50% B; 69–70 min, 50–100% B; 70–75 min, 100% B).

Raw data was searched by Skyline-daily (4.2.1.19004, University of Washington, USA). The proteins were quantified with the fragment total area reported by Skyline-daily. We selected peptides and tested their stability of signal and shape of peaks in the pool sample for final quantification, and referred to the ranking offered by skyline.

### Supplementary Information

**Additional file 1: **Supplementary figures.

**Additional file 2: Table S1**. Metadata.

**Additional file 3: Table S2.** Upregulated proteins.

**Additional file 4: Table S3.** Downregulated proteins.

**Additional file 5: Table S4.** Absolute quantification of the QconCAT proteins.

**Additional file 6: Table S5.** Dilution of QconCAT proteins.

**Additional file 7: Table S6.** Absolute quantification of the Quartet.

**Additional file 8: Table S7.** Targeted peptides.

**Additional file 9. **Review history.

## Data Availability

All proteomic raw data have been deposited to the ProteomeXchange Consortium (http://proteomecentral.proteomexchange.org) via the iProX [60, 61] partner repository with the dataset identifier PXD043262 [62]. In addition, all proteomics raw data have also been deposited at the Firmiana [33] platform (a one-stop proteomic cloud platform; https://phenomics.fudan.edu.cn), and the qualified profiling datasets were processed at the platform. All R scripts used for statistical analyses have been publicly available on GitHub: https://github.com/ecnuzdd/MAQC/tree/v1.0.0 (under the GNU General Public License v3.0) [63] and Zenodo: http://doi.org/10.5281/zenodo.8089593 [64].
