# Supplementary material for: Quartet protein reference materials and datasets for multi-platform assessment of label-free proteomics
Source: Genome Biol. 2023 Sep 7;24:202. doi: 10.1186/s13059-023-03048-y (PMC10483797; doi:10.1186/s13059-023-03048-y)
Supplement: Supplementary file 1 — Additional file 1: Supplementary figures. [file 13059_2023_3048_MOESM1_ESM.pdf]

Fig. S1

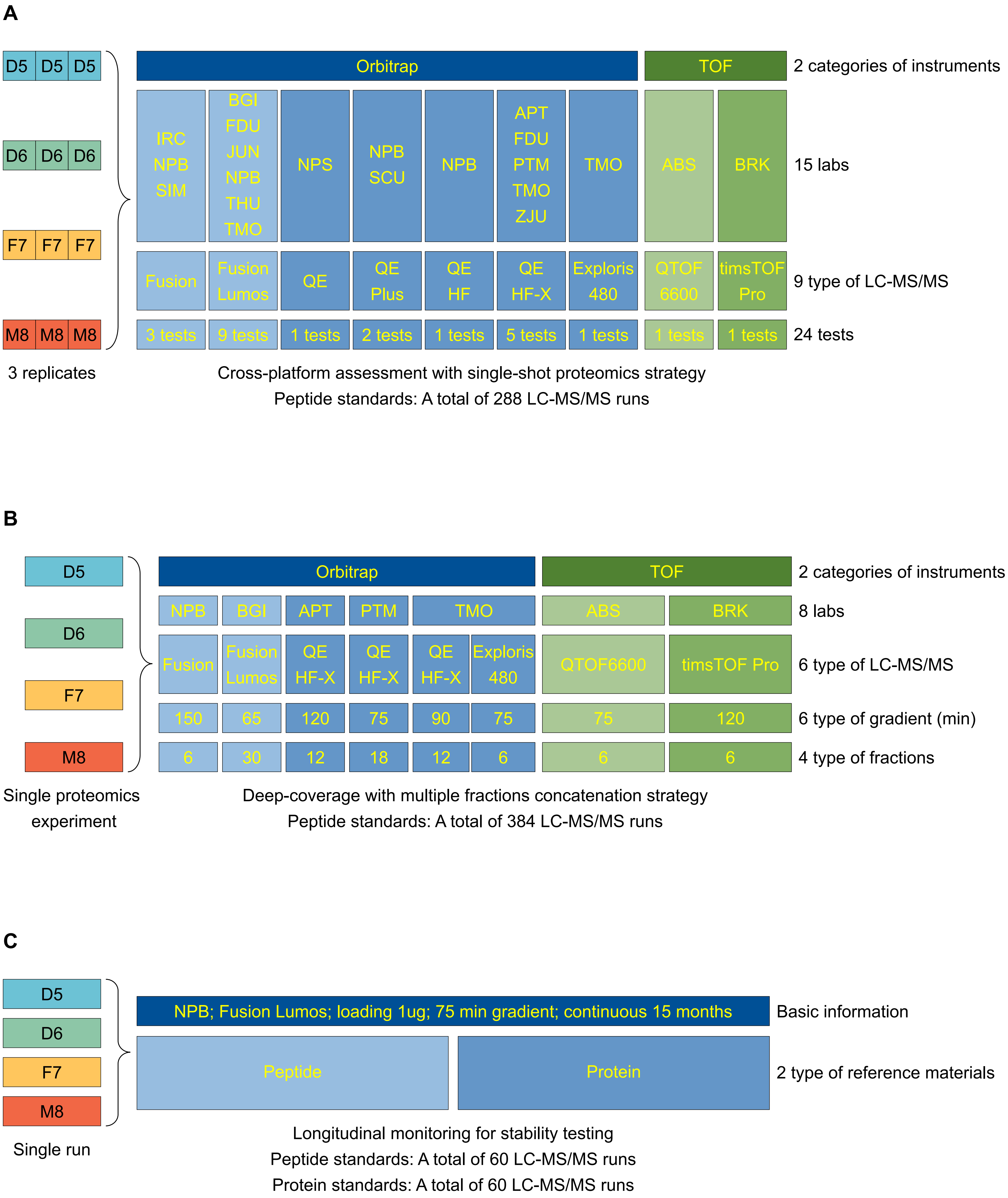
A
D5 D5 D5
D6 D6 D6
F7 F7 F7
M8 M8 M8
3 replicates
Orbitrap
TOF
2 categories of instruments
IRC NPB SIM
BGI FDU JUN NPB THU TMO
NPS
NPB SCU
NPB
APT FDU PTM TMO ZJU
TMO
ABS
BRK
15 labs
Fusion
Fusion Lumos
QE
QE Plus
QE HF
QE HF-X
Exploris 480
QTOF 6600
timsTOF Pro
9 type of LC-MS/MS
3 tests
9 tests
1 tests
2 tests
1 tests
5 tests
1 tests
1 tests
1 tests
24 tests
Cross-platform assessment with single-shot proteomics strategy
Peptide standards: A total of 288 LC-MS/MS runs
B
D5
D6
F7
M8
Single proteomics experiment
Orbitrap
TOF
2 categories of instruments
NPB
BGI
APT
PTM
TMO
ABS
BRK
8 labs
Fusion
Fusion Lumos
QE HF-X
QE HF-X
QE HF-X
Exploris 480
QTOF6600
timsTOF Pro
6 type of LC-MS/MS
150
65
120
75
90
75
75
120
6 type of gradient (min)
6
30
12
18
12
6
6
6
4 type of fractions
Deep-coverage with multiple fractions concatenation strategy
Peptide standards: A total of 384 LC-MS/MS runs
C
D5
D6
F7
M8
Single run
NPB; Fusion Lumos; loading 1ug; 75 min gradient; continuous 15 months
Basic information
Peptide
Protein
2 type of reference materials
Longitudinal monitoring for stability testing
Peptide standards: A total of 60 LC-MS/MS runs
Protein standards: A total of 60 LC-MS/MS runs

Fig. S2

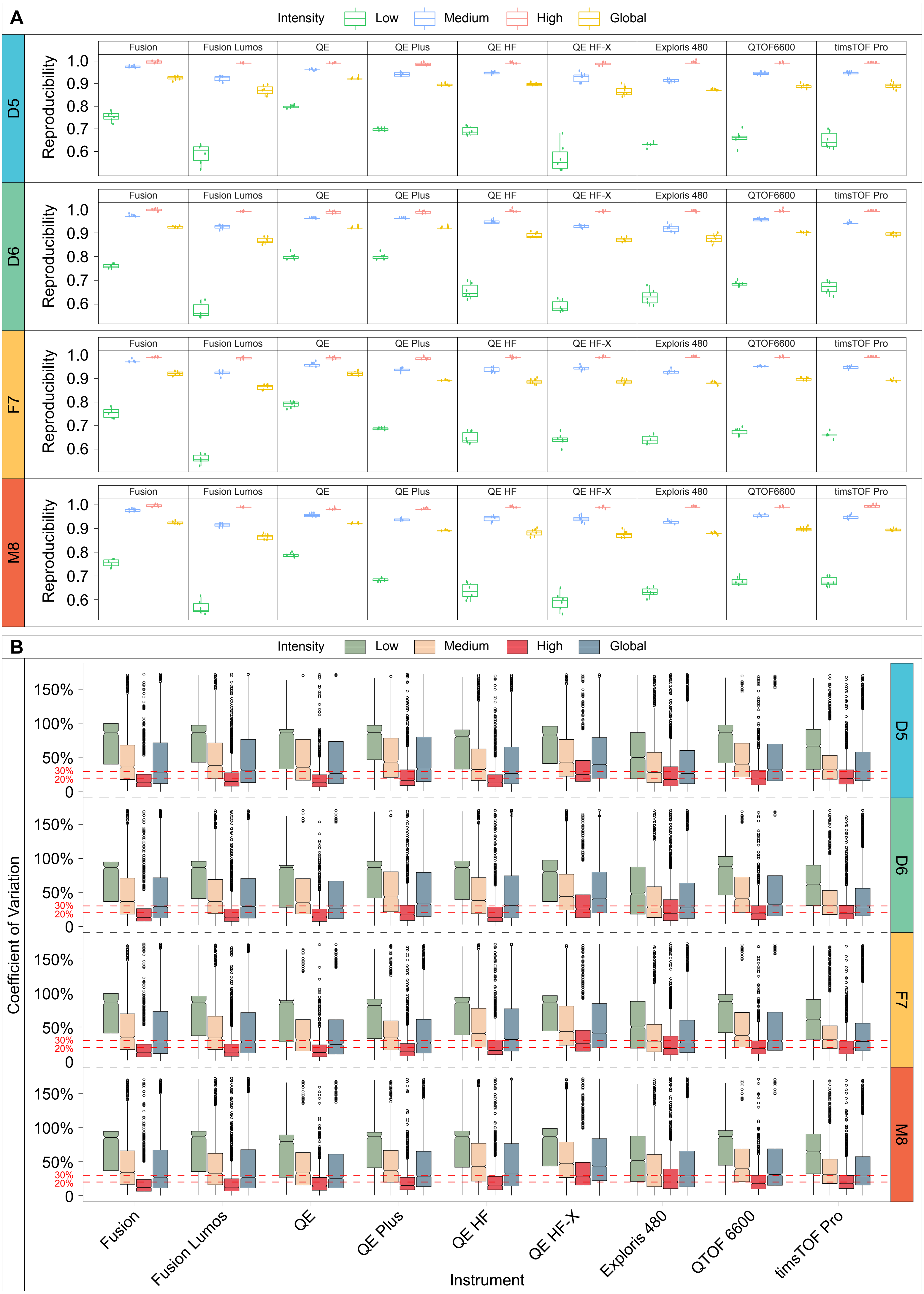

A
Intensity
Low
Medium
High
Global
D5
D6
F7
M8
Reproducibility
1.0
0.9
0.8
0.7
0.6
Fusion
Fusion Lumos
QE
QE Plus
QE HF
QE HF-X
Exploris 480
QTOF6600
timsTOF Pro
B
Intensity
Low
Medium
High
Global
Coefficient of Variation
150%
100%
50%
30%
20%
0
Fusion
Fusion Lumos
QE
QE Plus
QE HF
QE HF-X
Exploris 480
QTOF 6600
timsTOF Pro
Instrument

Fig. S3

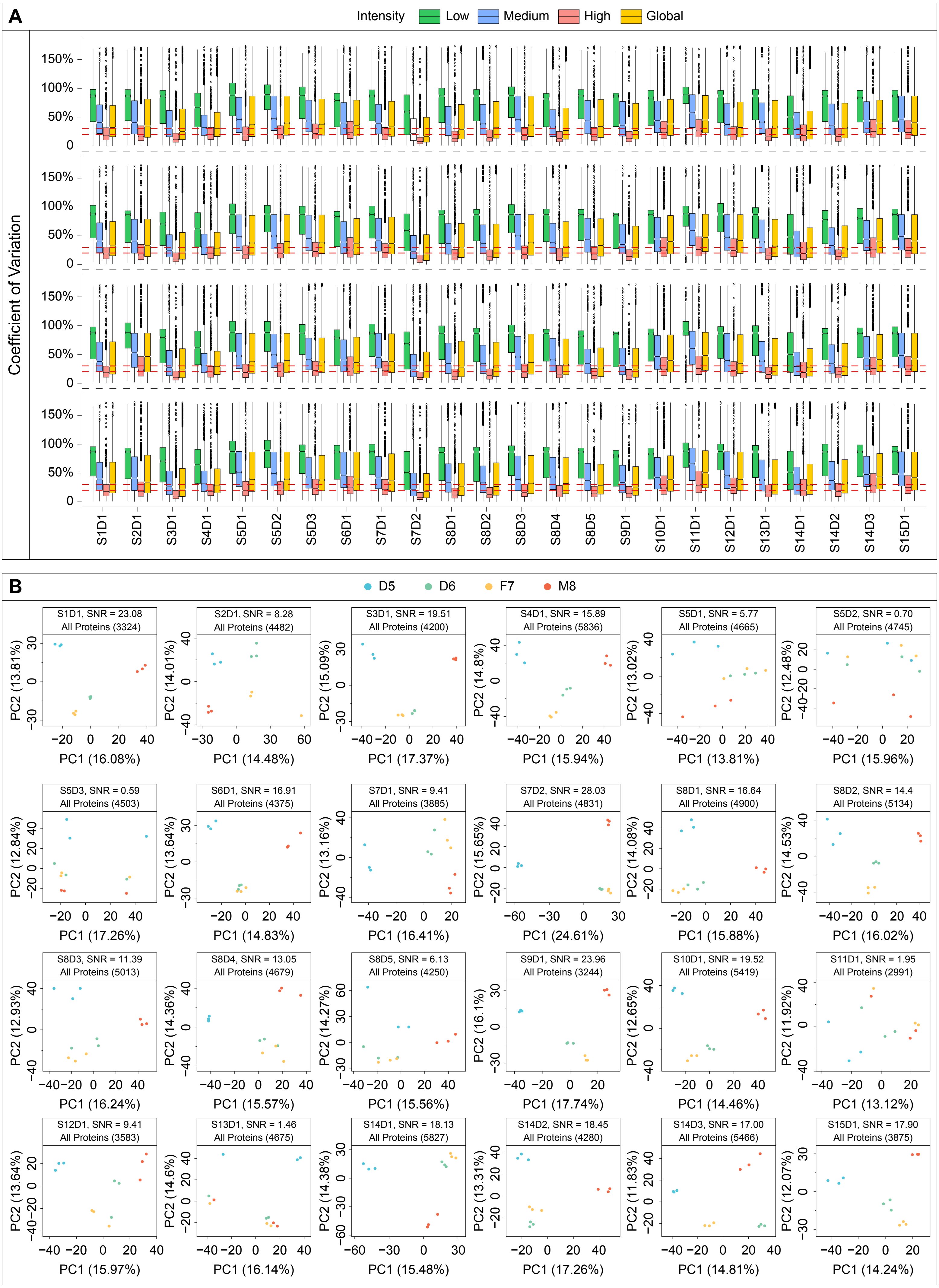
A
Intensity
Low
Medium
High
Global
Coefficient of Variation
150%
100%
50%
0
S1D1
S2D1
S3D1
S4D1
S5D1
S5D2
S5D3
S6D1
S7D1
S7D2
S8D1
S8D2
S8D3
S8D4
S8D5
S9D1
S10D1
S11D1
S12D1
S13D1
S14D1
S14D2
S14D3
S15D1
B
D5
D6
F7
M8
S1D1, SNR = 23.08
All Proteins (3324)
PC2 (13.81%)
PC1 (16.08%)
S2D1, SNR = 8.28
All Proteins (4482)
PC2 (14.01%)
PC1 (14.48%)
S3D1, SNR = 19.51
All Proteins (4200)
PC2 (15.09%)
PC1 (17.37%)
S4D1, SNR = 15.89
All Proteins (5836)
PC2 (14.8%)
PC1 (15.94%)
S5D1, SNR = 5.77
All Proteins (4665)
PC2 (13.02%)
PC1 (13.81%)
S5D2, SNR = 0.70
All Proteins (4745)
PC2 (12.48%)
PC1 (15.96%)
S5D3, SNR = 0.59
All Proteins (4503)
PC2 (12.84%)
PC1 (17.26%)
S6D1, SNR = 16.91
All Proteins (4375)
PC2 (13.64%)
PC1 (14.83%)
S7D1, SNR = 9.41
All Proteins (3885)
PC2 (13.16%)
PC1 (16.41%)
S7D2, SNR = 28.03
All Proteins (4831)
PC2 (15.65%)
PC1 (24.61%)
S8D1, SNR = 16.64
All Proteins (4900)
PC2 (14.08%)
PC1 (15.88%)
S8D2, SNR = 14.4
All Proteins (5134)
PC2 (14.53%)
PC1 (16.02%)
S8D3, SNR = 11.39
All Proteins (5013)
PC2 (12.93%)
PC1 (16.24%)
S8D4, SNR = 13.05
All Proteins (4679)
PC2 (14.36%)
PC1 (15.57%)
S8D5, SNR = 6.13
All Proteins (4250)
PC2 (14.27%)
PC1 (15.56%)
S9D1, SNR = 23.96
All Proteins (3244)
PC2 (16.1%)
PC1 (17.74%)
S10D1, SNR = 19.52
All Proteins (5419)
PC2 (12.65%)
PC1 (14.46%)
S11D1, SNR = 1.95
All Proteins (2991)
PC2 (11.92%)
PC1 (13.12%)
S12D1, SNR = 9.41
All Proteins (3583)
PC2 (13.64%)
PC1 (15.97%)
S13D1, SNR = 1.46
All Proteins (4675)
PC2 (14.6%)
PC1 (16.14%)
S14D1, SNR = 18.13
All Proteins (5827)
PC2 (14.38%)
PC1 (15.48%)
S14D2, SNR = 18.45
All Proteins (4280)
PC2 (13.31%)
PC1 (17.26%)
S14D3, SNR = 17.00
All Proteins (5466)
PC2 (11.83%)
PC1 (14.81%)
S15D1, SNR = 17.90
All Proteins (3875)
PC2 (12.07%)
PC1 (14.24%)

Fig. S4

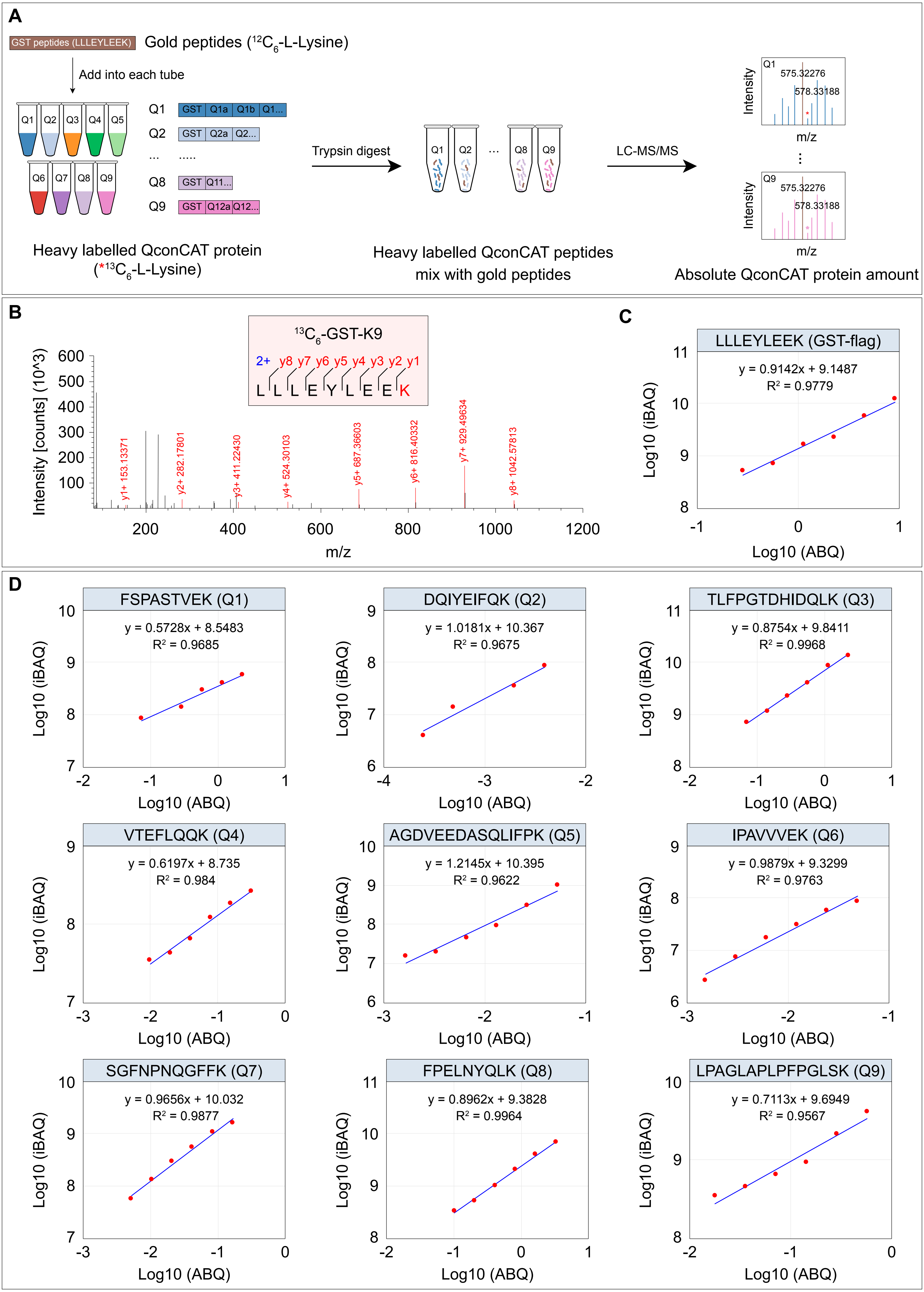
A
GST peptides (LLLEYLEEK) Gold peptides (12C6-L-Lysine)
Add into each tube
Q1 Q2 Q3 Q4 Q5 Q6 Q7 Q8 Q9
Q1 GST Q1a Q1b Q1...
Q2 GST Q2a Q2...
Q8 GST Q11...
Q9 GST Q12a Q12...
Heavy labelled QconCAT protein (*13C6-L-Lysine)
Trypsin digest
Heavy labelled QconCAT peptides mix with gold peptides
LC-MS/MS
575.32276
578.33188
Intensity
m/z
Absolute QconCAT protein amount
B
13C6-GST-K9
2+ y8 y7 y6 y5 y4 y3 y2 y1
L L L E Y L E E K
Intensity [counts] (10^3)
y1+ 153.13371
y2+ 282.17801
y3+ 411.22430
y4+ 524.30103
y5+ 687.36603
y6+ 816.40332
y7+ 929.49634
y8+ 1042.57813
m/z
C
LLLEYLEEK (GST-flag)
y = 0.9142x + 9.1487
R² = 0.9779
Log10 (iBAQ)
Log10 (ABQ)
D
FSPASTVEK (Q1)
y = 0.5728x + 8.5483
R² = 0.9685
DQIYEIFQK (Q2)
y = 1.0181x + 10.367
R² = 0.9675
TLFPGTDHIDQLK (Q3)
y = 0.8754x + 9.8411
R² = 0.9968
VTEFLQQK (Q4)
y = 0.6197x + 8.735
R² = 0.984
AGDVEEDASQLIFPK (Q5)
y = 1.2145x + 10.395
R² = 0.9622
IPAVVVEK (Q6)
y = 0.9879x + 9.3299
R² = 0.9763
SGFNPNQGFFK (Q7)
y = 0.9656x + 10.032
R² = 0.9877
FPELNYQLK (Q8)
y = 0.8962x + 9.3828
R² = 0.9964
LPAGLAPLPFPGLSK (Q9)
y = 0.7113x + 9.6949
R² = 0.9567
Log10 (iBAQ)
Log10 (ABQ)

Fig. S5

A

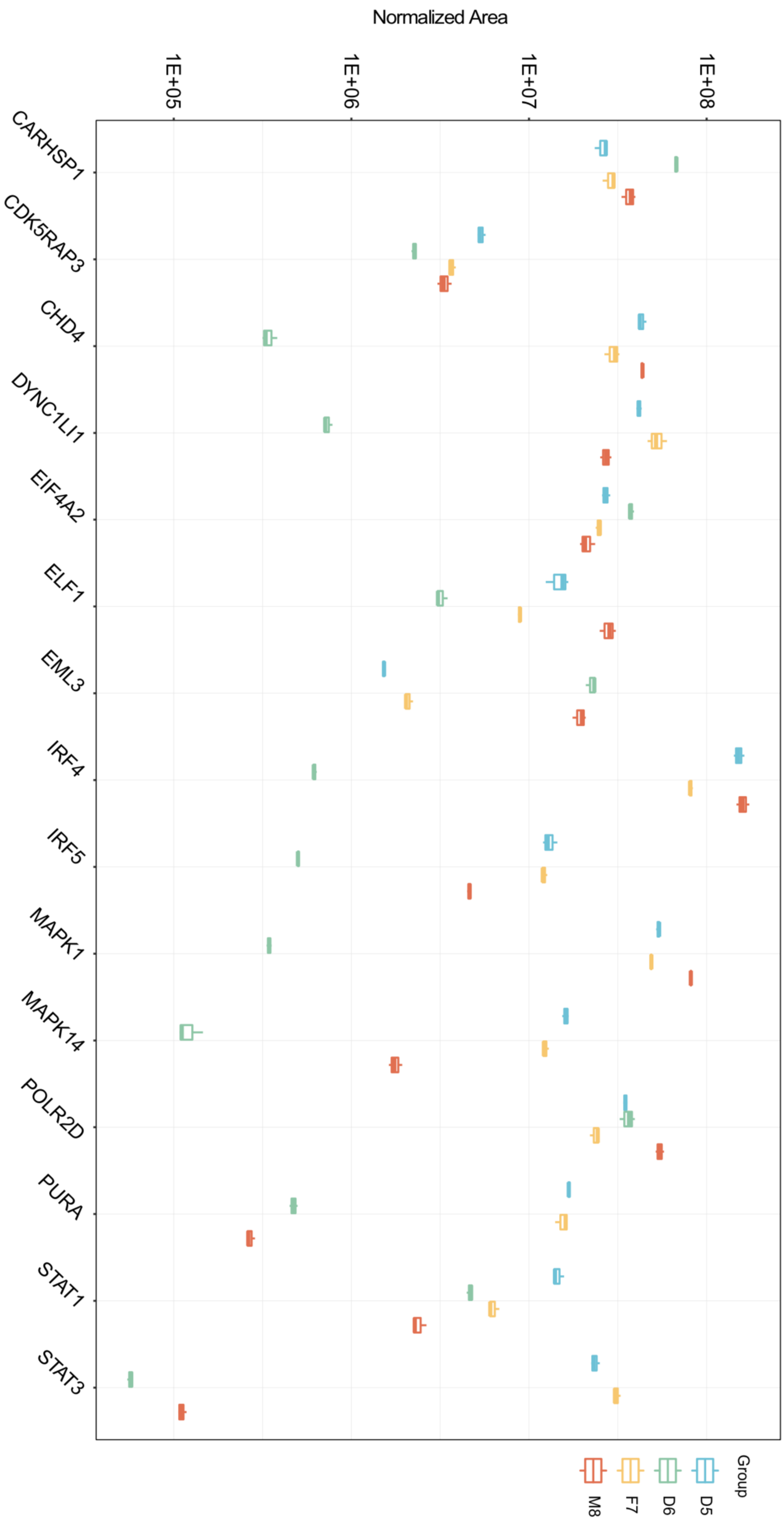
Normalized Area
1E+05
1E+06
1E+07
1E+08
CARHSP1
CDK5RAP3
CHD4
DYNC1LI1
EIF4A2
ELF1
EML3
IRF4
IRF5
MAPK1
MAPK14
POLR2D
PURA
STAT1
STAT3
Group
D5
D6
F7
M8

Fig. S6

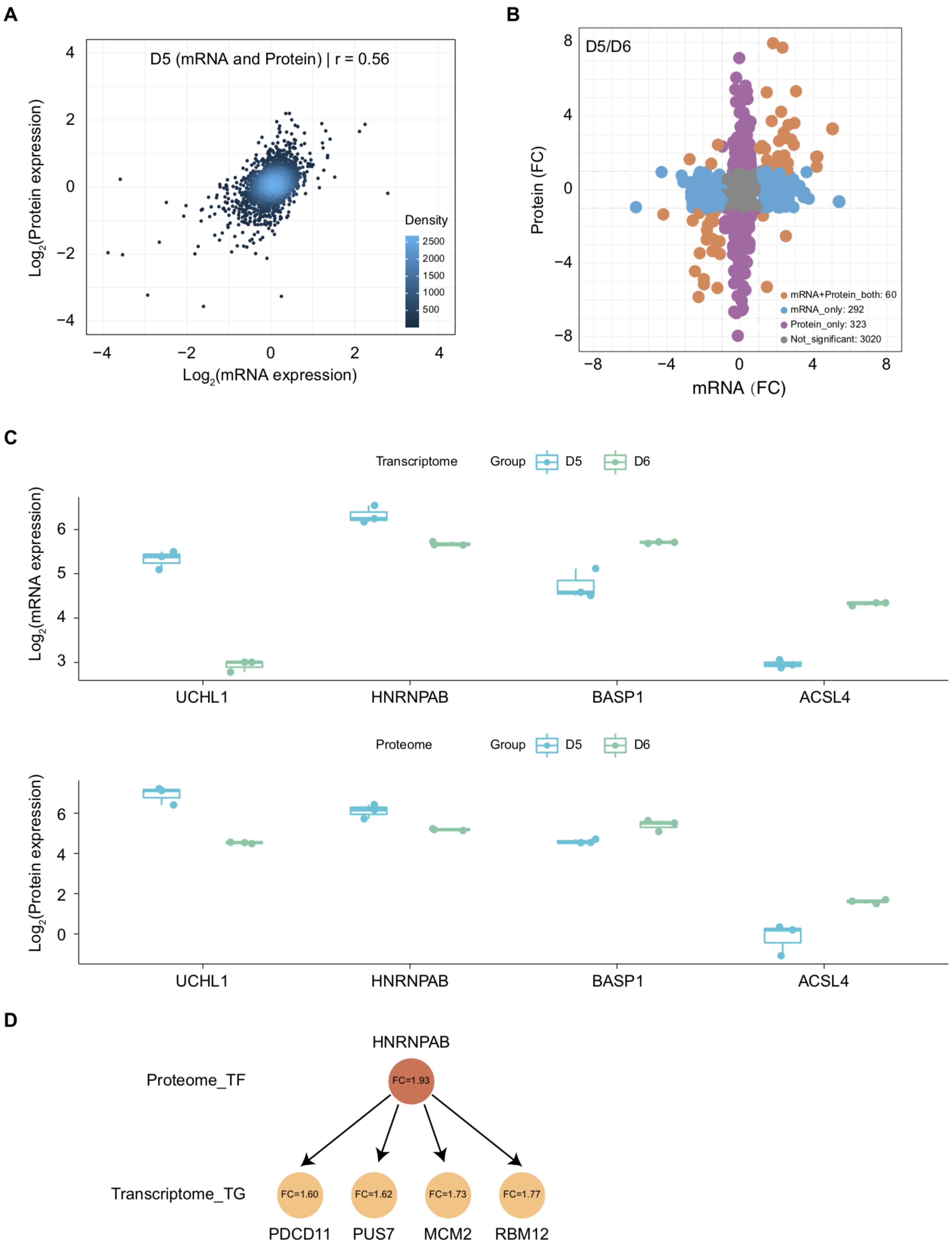

A
D5 (mRNA and Protein) | r = 0.56
Log2(Protein expression)
Log2(mRNA expression)
Density
2500
2000
1500
1000
500
B
D5/D6
Protein (FC)
mRNA (FC)
mRNA+Protein_both: 60
mRNA_only: 292
Protein_only: 323
Not_significant: 3020
C
Transcriptome
Group
D5
D6
Log2(mRNA expression)
UCHL1
HNRNPAB
BASP1
ACSL4
Proteome
Log2(Protein expression)
D
HNRNPAB
Proteome_TF
FC=1.93
Transcriptome_TG
FC=1.60
FC=1.62
FC=1.73
FC=1.77
PDCD11
PUS7
MCM2
RBM12

Fig. S7

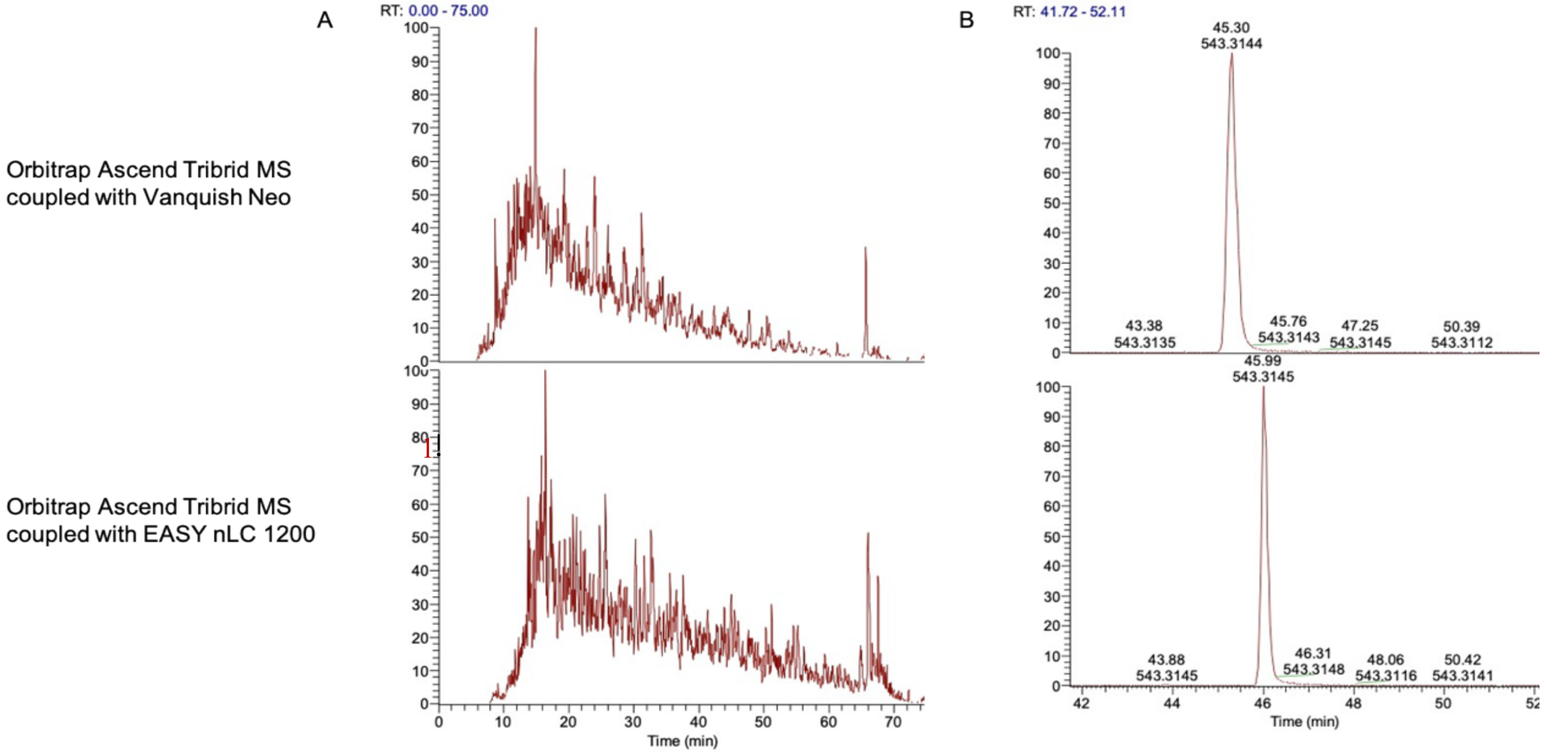
A
B
Orbitrap Ascend Tribrid MS coupled with Vanquish Neo
Orbitrap Ascend Tribrid MS coupled with EASY nLC 1200
RT: 0.00 - 75.00
RT: 41.72 - 52.11
45.30
543.3144
43.38
543.3135
45.76
543.3143
47.25
543.3145
50.39
543.3112
45.99
543.3145
43.88
543.3145
46.31
543.3148
48.06
543.3116
50.42
543.3141
Time (min)
Time (min)

Supplemental figure legends

Fig. S1 | MS files (N = 792) used to generate standard reference material datasets. A, Cross-platform assessment with the single-shot proteomics strategy (peptide standards with a total of 288 LC-MS/MS runs). **B**, Deep coverage with a multiple fraction concatenation strategy (peptide standards with a total of 384 LC-MS/MS runs). **C**, Longitudinal monitoring for stability testing (peptide standards: total of 60 LC-MS/MS runs; protein standards: total of 60 LC-MS/MS runs).

Fig. S2 | Quantitative reproducibility and variation analysis among nine conventional instruments. A, Reproducibility of detected proteins from a quantitative perspective (low-intensity, medium-intensity, high-intensity, and global groups) analyzed by nine conventional instruments in each sample of the Quartet. **B**, Quantitative variation of each sample of the Quartet in the low-intensity, medium-intensity, high-intensity, and global groups of nine conventional instruments (to facilitate overall comparison, the CVs of D5 among nine conventional instruments are repeated here).

Fig. S3 | Quantitative evaluation based on CVs and SNR. A, Quantitative variation of each sample of the Quartet in the low-intensity, medium-intensity, high-intensity, and global groups of 24 datasets produced by nine types of mass spectrometers across 15 laboratories (to facilitate overall comparison, the CVs of D5 among S8D2 and S3D1 are repeated here). **B**, Principal component analysis and SNR scoring of all proteins groups from 24 datasets produced by nine types of mass spectrometers across 15 laboratories (to facilitate overall comparison, the principal component analysis and SNR scoring in S8D2 and S3D1 are also repeated here).

Fig. S4 | Absolute quantification of the QconCAT proteins. A, Workflows for absolute quantification (ABQ) of the QconCAT proteins. **B**, MS/MS spectra (left panel) and dilution response curve (right panel) of the C13-labeled gold peptide (LLLEYLEEK) in GST-flag of the QconCAT proteins. **C**, Dilution response curve of the C13-labeled representative anchor peptide in every QconCAT protein.

Fig. S5 | Boxplot showing the expression of "anchor" proteins validated by PRM assay.

Fig. S6 | Analysis of transcriptome and proteome. A, the Pearson correlation coefficient between the transcriptome and proteome of the D5 sample is 0.56, which is consistent with previous research

findings (r=0.36~0.60). **B**, Gene products (GPs) expression pattern in D5 vs D6 at the transcriptome and proteome level. A total of 3,695 GPs were identified in D5 and D6, 292 GPs and 323 GPs were differentially expressed between D5 and D6, at the transcriptome and proteome levels, respectively. In which, 60 GPs were significantly differentially expressed on both transcriptome and proteome levels. **C**, The expression of UCHL1, HNRNPAB, BASP1, and ACSL4 in D5 and D6 at the transcriptome and proteome level (transcriptome level, top panel; proteome level, bottom panel). **D**, HNRNPAB and its target genes (PDCD11, PUS7, MCM2 and RBM12) in D5 sample were upregulated on both proteome and transcriptome levels, respectively.

Fig. S7 | Two different types of chromatographic instruments coupled with Ascend MS instrument for the detection of Quartet D5 samples. A, The total ion chromatogram (TIC) of the detection results of two different types of chromatographic instruments. **B**, The RT of the peptide of two different types of chromatographic instruments.
