# Supplementary material for: Quartet protein reference materials and datasets for multi-platform assessment of label-free proteomics
Source: Genome Biol. 2023 Sep 7;24:202. doi: 10.1186/s13059-023-03048-y (PMC10483797; doi:10.1186/s13059-023-03048-y)
Supplement: Supplementary file 9 — Additional file 9. Review history. [file 13059_2023_3048_MOESM9_ESM.docx]

Review History

**First round of review**

**Reviewer 1**

**Were you able to assess all statistics in the manuscript, including the appropriateness of statistical tests used?**

Yes, and I have assessed the statistics in my report.

**Comments to author:**

Tian and colleagues present results from a multi-site and multi-platform benchmarking effort to study the protein identification and quantification reproducibility. Biological material from four family members were distributed to 15 laboratories in China for label-free proteomics measurements, after which the bioinformatics analysis was performed in a central location.

Reproducibility of proteomics experiments is a relevant topic, although the authors present the situation much more direly than it actually is by stating that there has been "no investigation [...] for the proteomics community to systematically evaluate data reproducibility across laboratories" (p. 4). This statement is incorrect, as several such large-scale studies have been performed up to more than a decade ago. Unfortunately, the authors do not properly acknowledge previous studies in the manuscript. A few relevant papers include:

- "Repeatability and reproducibility in proteomic identifications by liquid chromatography-tandem mass spectrometry" (http://pubs.acs.org/doi/abs/10.1021/pr9006365): This study measured the reproducibility on multiple instruments at multiple sites in the context of the NCI CPTAC project. It is briefly mentioned in the Discussion but not properly acknowledged.

- "The 2012/2013 ABRF Proteomic Research Group Study: Assessing longitudinal intralaboratory variability in routine peptide liquid chromatography tandem mass spectrometry analyses" (http://www.mcponline.org/lookup/doi/10.1074/mcp.O115.051888): This study performed by the ABRF-PRG evaluated the longitunal variability across multiple laboratories.

- "Multicenter experiment for quality control of peptide-centric LC-MS/MS analysis — A longitudinal performance assessment with nLC coupled to orbitrap MS analyzers" (http://linkinghub.elsevier.com/retrieve/pii/S1874391915300105): This study performed longitudinal quality assessment anc quality control across multiple locations.

- "Reproducibility of differential proteomic technologies in CPTAC fractionated xenografts" (http://pubs.acs.org/doi/abs/10.1021/acs.jproteome.5b00859): This study performed an evaluation of variability in label-free and iTRAQ quantification results.

- "Signatures for mass spectrometry data quality" (http://pubs.acs.org/doi/abs/10.1021/pr401143e): This study performed an in-depth longitudinal analysis of QC samples and potential sources of variation.

- "The Human Proteome Organization-Proteomics Standards Initiative Quality Control Working Group: Making quality control more accessible for biological mass spectrometry" (http://pubs.acs.org/doi/abs/10.1021/acs.analchem.6b04310): There are existing efforts, such as the Proteomics Standards Initiative Quality Control Working Group.

Additionally, several other benchmarking datasets exist, such as:

- "A multicenter study benchmarks software tools for label-free proteome quantification" (http://www.nature.com/doifinder/10.1038/nbt.3685)

- "A comprehensive LFQ benchmark dataset on modern day acquisition strategies in proteomics" (https://www.nature.com/articles/s41597-022-01216-6)

These are only some of the most obvious examples of studies that have investigated similar aspects and provided similar conclusions as presented in the current work, but which have not been described. There are even more studies on the reproducibility of MRM, etc., that I did not mention here.

A fundamental aspect of performing a benchmarking study is that it can be independently repeated by other researchers. Unfortunately this is not possible with the Quartet benchmark. There is no information how and where researchers can acquire the Quartet samples. The Methods are lacking all but the most basic information, providing no details on what was done. What were the SOPs for the different instrument platforms? How was the bioinformatics analysis performed? For example, "Peptide quantification was performed according to the peak area," is the full extent to which label-free quantification is described, despite this being a major focus of the manuscript and advertised in the title. At the very least full information should be provided that allows other researchers to recreate this analysis, including software name, version, and detailed steps. Better would be if the analysis code was publicly available on e.g. GitHub. MS/MS data is not available either. It is mentioned that it has been uploaded to iPROX, but the advertised dataset number does not exist or is possibly still private. In any case, I could not find the data.

Other experimental details are also missing or only vaguely described. For example, the SNR calculation is only detailed in Figure 4E, but is not even described in the Methods. Relevant related literature is again not described either, such as "QC metrics from CPTAC raw LC-MS/MS data interpreted through multivariate statistics" (http://pubs.acs.org/doi/abs/10.1021/ac4034455) which used PCA for quality control and to interpret sources of variability.

In light of the fact that there are large omissions of the literature, and that previous studies have been more comprehensive and already produced the same conclusions, unfortunately I cannot recommend publication of the current manuscript.

**Reviewer 2**

**Were you able to assess all statistics in the manuscript, including the appropriateness of statistical tests used?**

Yes, and I have assessed the statistics in my report.

**Comments to author:**

The authors present the generation and proteomic characterization of a human standard reference material (referred to as Quartet) for the qualitative and quantitative evaluation of reproducibility and reliability of different MS-based proteomics strategies, instruments and laboratories. In addition, it enables the identification of potential variation sources and provides resources for development and optimization as well as they validated the stability of the material for at least one year.

The characterization of the Quartet is very well designed and included various parameters (e.g. identification numbers, intensity, gene ontology annotation, confidence intervals, fraction-of-total, signal-to-noise-ratio). The results are nicely presented and are very exciting and promising. The manuscript is well written with concise and understandable formulations.

I only have a few suggestions/questions:

* When comparing and discussing the evaluation of different MS instrument performances, maybe the authors could also mention and discuss the impact and significance of the coupled chromatographic system. To my understanding, these chromatographic systems were not uniform across the different laboratories. Thus, they definitely could influence the considered parameters of identification numbers, etc. and consequently additionally influence the overall performance, which makes a direct comparison of explicit MS instrument performances difficult.

* In figure 1, the last subsection should be "C" instead of "D".

* Could the authors please mention how the used lymphoblastoid cells, used as basis for the generated Quartet material, were immortalized?

* Could the authors provide an outlook / test-case, how the Quartet could be used and integrated into potential translational/clinical studies as mentioned in the manuscript and how its usage could improve the studies´ quality?

* The authors provide "anchor" proteins for potential calibration as well as "indicator" proteins to evaluate the quantitative reliability of different MS platforms. To corroborate their findings of anchor and indicator proteins, the authors could exemplarily validate the observed protein expression results with a complementary method, like targeted mass spectrometry or an antibody-dependent method.

**Authors Response**

**Point-by-point responses to the reviewers’ comments:**

GBIO-D-22-01638

**Quartet protein reference materials and datasets for multi-platform assessment of label-free proteomics**

**REVIEWER COMMENTS**

Reviewer #1 (Remarks to the Author):

Tian and colleagues present results from a multi-site and multi-platform benchmarking effort to study the protein identification and quantification reproducibility. Biological material from four family members were distributed to 15 laboratories in China for label-free proteomics measurements, after which the bioinformatics analysis was performed in a central location.

Q1. Reproducibility of proteomics experiments is a relevant topic, although the authors present the situation much more direly than it actually is by stating that there has been "no investigation [...] for the proteomics community to systematically evaluate data reproducibility across laboratories" (p. 4). This statement is incorrect, as several such large-scale studies have been performed up to more than a decade ago. Unfortunately, the authors do not properly acknowledge previous studies in the manuscript. A few relevant papers include:

- "Repeatability and reproducibility in proteomic identifications by liquid chromatography-tandem mass spectrometry" (http://pubs.acs.org/doi/abs/10.1021/pr9006365): This study measured the reproducibility on multiple instruments at multiple sites in the context of the NCI CPTAC project. It is briefly mentioned in the Discussion but not properly acknowledged.

- "The 2012/2013 ABRF Proteomic Research Group Study: Assessing longitudinal intralaboratory variability in routine peptide liquid chromatography tandem mass spectrometry analyses" (http://www.mcponline.org/lookup/doi/10.1074/mcp.O115.051888): This study performed by the ABRF-PRG evaluated the longitunal variability across multiple laboratories.

- "Multicenter experiment for quality control of peptide-centric LC-MS/MS analysis — A longitudinal performance assessment with nLC coupled to orbitrap MS analyzers" (http://linkinghub.elsevier.com/retrieve/pii/S1874391915300105): This study performed longitudinal quality assessment and quality control across multiple locations.

- "Reproducibility of differential proteomic technologies in CPTAC fractionated xenografts" (http://pubs.acs.org/doi/abs/10.1021/acs.jproteome.5b00859): This study performed an evaluation of variability in label-free and iTRAQ quantification results.

- "Signatures for mass spectrometry data quality" (http://pubs.acs.org/doi/abs/10.1021/pr401143e): This study performed an in-depth longitudinal analysis of QC samples and potential sources of variation.

- "The Human Proteome Organization-Proteomics Standards Initiative Quality Control Working Group: Making quality control more accessible for biological mass spectrometry" (http://pubs.acs.org/doi/abs/10.1021/acs.analchem.6b04310): There are existing efforts, such as the Proteomics Standards Initiative Quality Control Working Group.

Additionally, several other benchmarking datasets exist, such as:

- "A multicenter study benchmarks software tools for label-free proteome quantification" (http://www.nature.com/doifinder/10.1038/nbt.3685)

- "A comprehensive LFQ benchmark dataset on modern day acquisition strategies in proteomics" (https://www.nature.com/articles/s41597-022-01216-6)

These are only some of the most obvious examples of studies that have investigated similar aspects and provided similar conclusions as presented in the current work, but which have not been described. There are even more studies on the reproducibility of MRM, etc., that I did not mention here.

**Response:** We appreciate the reviewer's scientific rigor and constructive comments. We agree with the reviewer that several such large-scale studies have been performed up to more than a decade ago for the reproducibility of proteomics experiments. We response this comment from the following two aspects: summary of previous work and five distinct characteristics of our study.

**1) Summary of previous work**

Previous researchers did make great efforts in the reproducibility evaluation of proteomics experiments and the establishment of benchmark datasets. Paulovich et al. put forward a standard operating procedure (SOP) for the large-scale production of yeast standards and offered aliquots to the proteomics community through the National Institute of Standards and Technology (NIST), where the yeast proteome is under development as a certified reference material to meet the long-term needs of the community[1] **(*Mol Cell Proteomics*, 2009, PMID: 19858499)**. Bell et al. distributed an equimolar test sample comprising 20 highly purified recombinant human proteins to different laboratories. The centralized analysis determined missed identification (false negatives), environmental contamination, database matching, and curation of protein identification as sources of irreproducibility in LC-MS/MS-based proteomics[2] **(*Nat Methods*, 2009, PMID: 19448641)**. Tabb et al. employed protein mixtures to measure the repeatability and reproducibility on multiple instruments (four Thermo LTQ and four Orbitrap instruments) at 5 laboratories[3] **(*J Proteome Res*, 2010, PMID: 19921851)**. The Spanish proteomics laboratory network, ProteoRed, used identical yeast standard sample and similar mass spectrometry instrumentation to perform longitudinal quality assessment and quality control across 6 consortium proteomic platforms in a long-term fashion[4] **(*J Proteomics*, 2015, PMID: 25982386)**. The NCI Clinical Proteomic Tumor Analysis Consortium (CPTAC) employed a pair of reference xenograft proteomes for performing an evaluation of variability in label-free and iTRAQ quantification results[5] **(*J Proteome Res*, 2015, PMID: 26653538)**. The Association of Biomolecular Resource Facilities (ABRF-PRG) provided aliquots of a six bovine protein tryptic digest mixture every month for a period of nine months to systematically evaluate the longitudinal variability across multiple laboratories[6] **(*Mol Cell Proteomics*, 2015, PMID: 26435129)**. Amidan et al. built a classifier for automatically detecting out-of-control data sets, which could perform an in-depth longitudinal analysis of QC samples and potential sources of variation[7] **(*J Proteome Res*, 2014, 24611607)**. To determine the inter- and intra-laboratory reproducibility and performance of sequential window acquisition of all theoretical fragment ion spectra (SWATH)-MS for large-scale quantitative proteomics, Collins et al. created a benchmarking sample set comprising 30 stable isotope-labeled standard peptides diluted into a complex background, and then distributed the aliquots to 11 laboratories to assess the reproducibility of quantitative proteomic data. Using SWATH-MS data acquisition, the reproducibility, limit of detection, and linear dynamic range of quantitative characteristics were highly comparable across datasets from all sites[8] **(*Nat Commun*, 2017, PMID: 28827567)**. In order to make quality controlled more accessible for biological mass spectrometry, the Human Proteome Organization has established the Proteomics Standards Initiative Quality Control Working Group to provide a unified framework for quality control data[9] **(*Anal Chem*, 2017, PMID: 28318237)**. We summarized previous researches as follows **(See the Figure RL 1 for details)**:

a) Most of the previous studies distributed the aliquots (a single sample) to multiple laboratories for proteome identification. The defined mixtures of peptides or proteins can meet some benchmarking and QC needs, but in order to fully reflect the challenges of LC-MS/MS data acquisition in complex biological samples, more sophisticated reference materials are required.

b) Some studies made pairwise comparisons between samples. For example, the CPTAC used a pair of reference xenograft differential proteomes for initial platform validation and ongoing quality control on 6 instruments across 5 laboratories. However, due to the unsustainability of the PDX model as the source of the samples, it is difficult to promote on a large scale and to obtain official certification as a reference material.

c) Previous studies primarily evaluated mass spectrometry instruments that were mostly launched before the year 2015, including the LTQ and Q-Exactive. In the past decades, with the rapid development of LC-MS/MS, more advanced mass spectrometers, including Fusion Lumos, Q-Exactive HF-X, Exploris 480, and timsTOF pro, have become the backbone of scientific research. However, there has been relatively few researches conducted on quality control in related to these new version instruments.

**Figure RL 1** | Overview of the previous works related to our study.

**2) As an extension of previous studies, our work has the following five distinct characteristics:**

**a) Quartet family-based multi-omics study on the same sample**

Currently, multi-omics strategies have become a routine approach to obtain the molecular landscape of various types of tumors across multiple biological dimensions, which can help to elucidate novel molecular subtypes and abnormally activated signaling pathways, and find potential therapeutic targets. The Cancer Genome Atlas (TCGA) and Clinical Proteomic Tumor Analysis Consortium (CPTAC) have published landmark multi-omics studies, improving our cognition of brain cancer[10] **(*Cell*, 2020, PMID: 33242424)**, hepatocellular carcinoma[11] **(*Cell*, 2019, PMID: 31730861)**, lung cancer[12] **(*Cell*, 2020, PMID: 32649875)**, lung adenocarcinoma[13] **(*Cell*, 2020, PMID: 32649874;)**, colon cancer[14, 15] **(*Cell*, 2019, PMID: 31031003; *Nat Commun*, 2014, PMID: 25043054)**, clear cell renal cell carcinoma[16, 17] **(*Cell*, 2019, PMID: 31675502; *Nat Commun*, 2022, PMID: 35440542)**, endometrial carcinoma[18] **(*Cell*, 2020, PMID: 32059776)**, ovarian cancer[19] **(*Cell*, 2016, PMID: 27372738)**, and breast cancer[20, 21] **(*Nature*, 2016, PMID: 27251275; *Cell*, 2020, PMID: 33212010)** and so on. In these studies, a sample is usually divided into three (or four) parts so that DNA, RNA, and protein (or metabolites) can be extracted simultaneously during the sample preparation process, and then used for multi-omics data generation and integrated analysis.

Multi-omics research on tumors is in full swing, however, research on multi-omics reference materials for the same sample is seriously lagging behind. Therefore, multi-omics reference materials and relevant QC metrics from the same sample of interconnected reference materials are required for quality assessment of each omics measurement. To solve the unmet need, we launched the Quartet Project to provide multi-omics “ground truth” as well as best practices for the QC and data integration of multi-omics profiling. The Quartet multi-omics reference material suites included DNA, RNA, proteins, and metabolites developed from the immortalized cell lines derived from a quartet family of parents (F7 and M8) and monozygotic twin daughters (D5 and D6). Two types of “built-in truth” are provided for quality assessment of multi-omics profiling. One is the ability to correctly distinguish samples into four clusters (D5, D6, F7, and M8). For each omics type, their data generation and analysis must have the basic ability to differentiate the four different biological samples from technical replicates. The other one is the ability to correctly identify the hierarchical relationships across multi-omics features according to the rule of the central dogma, which can be used for assessing the reliability of correlation-based multi-omics network integration. For more details, see Figure RL 2. (Notably, our sister manuscript, which introduces MAQC-IV framework, has been revised in ***Nature Biotechnology***.).

**Figure RL 2** | Overview of the Quartet Project.

**a,** Design and production of Quartet family-based multi-omics reference material suites. **b,** Data generation across multiple platforms, labs, batches, and omics types. **c,** Workflow for the construction of reference datasets of differentially expressed features. Reference datasets were constructed according to the following steps: (1) Identifying detectable multi-omics features and per-sample normalization. (2) Intra-batch quality control. Features that were not detectable or had low technical reproducibility were filtered out. (3) Cross-batch quality control. Features detectable in a sufficient number of batches were retained. (4) Calculating intra-batch differentially expressed features (DEFs) using t-test analysis. DEFs were classified as up- or down-regulated based on the positive or negative sign of the log2 fold change. (5) Voting based on the regulatory directionality (up or down) to screen the high confidence DEFs.

Currently, in multi-omics projects, it is common practice to split a single sample into multiple parts for analysis across different omics platforms. Although the quality of each individual omics is controllable, it remains unclear whether the results across different omics datasets can be harmonized and used for integrated multi-omics analysis. Previous multi-omics projects mainly focused on quality control within each omics using only a single sample. In contrast, our work provides quality control samples consisting not only of four individuals but also encompassing four different omics types (genome, transcriptome, proteome, and metabolome), enabling cross-omics quality control in multi-omics projects. Here, we performed multi-omics analysis using transcriptomic and proteomic datasets as an example to illustrate the cross-omics ground truth generated by the Quartet reference material we developed. For example, the Pearson correlation coefficient between the transcriptome and proteome of the D5 sample is 0.56 **(Figure RL 3A)**, which is consistent with previous research findings (r=0.36~0.60)[22, 23] **(*Nat Biotechnol*, 2019, PMID: 30778230; *Cell*, 2016, PMID: 27104977)**. Furthermore, we analyzed gene products (GPs) expression pattern in different pairwise comparisons at the transcriptome and proteome level. D5 vs D6 was shown as an example. A total of 3,695 GPs were identified in D5 and D6, 292 GPs and 323 GPs were differentially expressed between D5 and D6, at the transcriptome and proteome levels, respectively. In which, 60 GPs were significantly differentially expressed on both transcriptome and proteome levels **(Figure RL 3B)**. We have also presented several genes that show consistent expression trends on both transcriptome and proteome levels, which are the ground truth of multi-omics analysis. For instance, UCHL1 and HNRNPAB were found to be upregulated in D5, while BASP1 and ACSL4 were upregulated in D6 on both transcriptome and proteome levels **(Figure RL 3C).**

Additionally, another advantage of using the Quartet samples for multi-omics quality control is that it can provide cross-omics interpretability. For instance, the changes in transcription factors (TFs) at the protein level may be related to the expression of their target genes at the transcriptional level. As illustrated by Figure RL 3D, HNRNPAB and its target genes (PDCD11, PUS7, MCM2 and RBM12) in D5 sample were higher than that in D6 on both proteome and transcriptome levels, respectively.

**Figure RL 3** | Analysis of transcriptome and proteome.

**A,** Correlation analysis of protein and transcript expression levels. **B,** Gene products (GPs) expression pattern in D5 vs D6 at the transcriptome and proteome level. **C,** The expression of UCHL1, HNRNPAB, BASP1, and ACSL4 in D5 and D6 at the transcriptome and proteome level (transcriptome level, top panel; proteome level, bottom panel). **D,** HNRNPAB and its target genes (PDCD11, PUS7, MCM2 and RBM12) in D5 sample were upregulated on both proteome and transcriptome levels, respectively.

**b) Quartet family-based inter- and intra- group different analysis through “SNR”**

Protein standards in previous studies were almost designed from a single proteomic and single sample source perspective. The most frequently used metrics are the repeatability and reproducibility in proteomic identifications, as well as coefficient of variation and correlation coefficient in proteomic quantification. These metrics only focus on within-group difference comparison from single sample but do not exhibit comparative proteomics difference in different biological samples.

In our study, the Quartet protein reference materials derived from a four-member Quartet family including two monozygotic twin daughters (D5 and D6) and their father (F7) and mother (M8), providing built-in truth defined by family relationship and the central dogma. Proteomic difference (built-in truth) from four individuals can be clearly depicted in two-dimensional (2-D) PCA plot. Four samples could be completely separated by the use of PCA, three replicates of each sample clustered compactly. Based on the Quartet sample design and their visualization in 2-D PCA plot, we can calculate the intrinsic biological differences (“signal”) among the Quartet samples and variations among technical replicates within the same sample group (“noise”). This allows us to define the "SNR" metric as the ratio between "signal" and "noise", which can gauge the performance of a platform, a lab, a protocol, or a batch. A higher SNR value indicates higher discriminating power and SNR value around or below zero means that it is almost impossible to distinguish different sample groups under the high level of technical noises **(Figure RL 4)**.

**Figure RL 4** | Signal-to-noise ratio enables assessment.

**a,** Concept of calculating signal-to-noise ratio (SNR). SNR was established to characterize the ability of a platform, a lab, or a batch to distinguish the intrinsic differences among distinct biological sample groups (“signal”) from variations in technical replicates of the same sample group (“noise”). **b,** Examples of good and bad batches with their SNR values and corresponding principal components analysis (PCA) scatter plots.

**c) Providing finely differentially expressed proteins (DEPs) with confidence interval.**

In addition to the advantages above, based on the Quartet sample design, the comparative proteomics performance of distinguishing built-in truth from different biological samples can be easily evaluated for multiple instruments or at multiple labs. Currently, protein reference datasets from single sample source mainly contain detection frequency and average expression. DEPs reference datasets remain lacking detailed differentiation.

The reference datasets based on intrinsic biological differences among the Quartet protein reference materials have been constructed and can be used as “ground truth” for quality assessment. We counted the occurrence frequency of up- and down-regulated DEPs in the 24 experiments by pairwise comparisons between samples within each experiment, and divided them into eight intervals according to the reproducibility frequency of DEPs as shown in Fig. 3c. These proteins can be used as a benchmark dataset to evaluate the quantitative ability of comparative proteomics on different platforms.

**d) Absolute quantification of the Quartet protein reference materials.**

To promote the large-scale, worldwide application of the Quartet as a proteome reference material, it is necessary to calculate the absolute number of the whole proteome (copy number per cell) according to the international metrological rules established by the International Organization for Standardization (ISO). To this end, we used the QconCAT[24] **(*J Proteome Res*, 2011, PMID: 21699228)** method to measure the internal standard “anchor” proteins. We randomly selected 33 proteins with intensity-based absolute quantiﬁcation (iBAQ) values distributed in four orders of magnitude as anchor proteins for absolute quantification and calibration of the copy numbers for the whole proteome. Representative unique peptides from these 33 anchor proteins were designed as QconCAT proteins by tandem fusion and split into 9 QconCAT proteins. Over 99% of the QconCAT proteins grown in the heavy isotope medium incorporated the labeled lysine, demonstrating the effectiveness of this approach.

To determine the absolute molar value of the C^13^-labeled QconCAT proteins, we synthesized the gold C^12^ GST peptide LLLEYLEEK (Institute of Metrology) and added gold peptides to nine tubes corresponding to the nine QconCAT proteins, which were digested with trypsin and loaded into the LC-MS/MS system for detection (Supplementary Fig. 4a). The molar amount of each QconCAT protein can then be calculated (Supplementary Table 4). For Quartet standard quantification, a dilution series of the C^13^-labeled QconCAT peptides was mixed with the Quartet peptide standard samples and then subjected to LC-MS/MS analysis (Fig. 8a). The corresponding heatmap portrays the linear trend of all identified C^13^-labeled anchor peptides (Fig. 8b and Supplementary Table 5). A dilution response curve of every C^13^-labeled anchor peptide (including GST peptide LLLEYLEEK) in the QconCAT proteins showed excellent linearity (Supplementary Fig. 4b, c), suggesting the good quantification accuracy of QconCAT proteins and the reliability of endogenous proteins in the Quartet anchored by QconCAT proteins.

The absolute molar value of the corresponding proteins in the Quartet standard sample was determined based on quantification of the C^13^-labeled anchor peptides (Supplementary Table 6). The copy number of the 33 anchor proteins ranged from 10^2^ to 10^6^ copies/cell. We aligned the absolute copy numbers (ABQ) of the anchor proteins to their corresponding iBAQ values and found that the ABQs and iBAQs were well-correlated (R^2^ = 0.7581-0.8052) (Fig. 8c), demonstrating consistency between relative quantification (iBAQ) and absolute quantification (copy number). Finally, we quantified the abundance of more than 4,000 proteins in each sample of the Quartet reference materials by aligning the proteome to the anchor proteins (Supplementary Table 6). Their dynamic ranges spanned over ~7 orders of magnitude from 10^1^ to 10^8^ copies/cell. Thus, absolute quantification of the Quartet proteome can provide reference datasets for QC and quality assessment of an LC-MS/MS platform for both basic research and clinical applications.

Furthermore, our reference materials are prepared and measured with a clearly defined traceability, and can be verified by multiple detection methods, ensuring the accuracy and reliability of the results.

**e) Proteome reference material establishment complying with the First Class of National Reference Materials will serve the proteomics community.**

The Quartet Project provides the community with multi-omics (Genomic, transcriptomic, proteomic and Metabolomic) reference materials and reference datasets of four samples from a family Quartet, which are 1:1 matched, for objectively assessing quality in data generation and integrated analysis in increasingly large-scale multi-omics studies. It is worth mentioning that the DNA and RNA reference material suites have been approved by China's State Administration for Market Regulation as the First Class of National Reference Materials (Standard Material Numbers: GBW0900-GBW0907) and are extensively being utilized for proficiency testing and method validation **(Figure RL 5)**. As a part of the Quartet Project for the quality control and data integration of multi-omics profiling, this Quartet Human B-lymphocyte proteomic protein standard reference materials are also in the national first-class reference materials approval process. After formal approval and release, the standard reference materials can not only be shared with various platforms, but also obtain standard reference datasets of materials through the “National Metrological Science Data Center” (https://srd.nmdc.ac.cn/gene/chinese-quartet/) for quality assessment between laboratories **(Figure RL 6)**. Overall, our series of research work provides unique multi-omics resources for evaluating the performance of labs, platforms, protocols, and analytical tools based on the pedigree information of the Quartet samples, and also provides unique insights into the selection of commonly used multi-omics integration strategies.

**Figure RL 5** | Summary of Quartet multi-omics reference materials.

**a,** Packaging of reference materials. **b,** Product packaging of reference materials. **c,** Storage of 1000 tubes of protein reference materials.

**Figure RL 6** | Quality Control and Data Integration of Multi-omics Profiling.

In summary, on the basis of the previous QC studies, we further developed multi-omics reference materials suites, which could be used for simultaneously evaluating between- and within-group differences based on the Quartet sample design containing pedigree information. In our reference datasets, “ground truth” of proteins not only contain detection frequency and average expression but also include their differential expression attributes. Cross-omics DEPs were also annotated. Additionally, for each protein in reference datasets, their relative and absolute quantification are provided. The Quartet protein reference materials are established by complying with the ISO, and have been publicly available at chinese-quartet.org, as well as currently in the process of being approved.

Our work is a crucial component of the MAQC-IV (framework introduction revised in ***Nat. Biotechnol***; DNA paper revised in ***Genome Biol***; RNA paper revised in ***Genome Biol***, Protein paper revised in ***Genome Biol*** and Metabolite paper revised in ***Genome Biol***), and provides the life science community with a unique and valuable proteome resource, which could serve as a reference for the research community to evaluate new technologies, labs, assays, products, lab operators, and computational algorithms. Additionally, this work not only advances the field of proteomic quality control research based on previous work, but also contributes the proteomics power to conduct high-quality, large-scale multi-omics studies. We strongly recommend the use of Quartet reference materials to optimize experimental methods and monitor the status of experimental platforms for producing high-quality data, and discovering more useful knowledge, thereby promoting the development of precision medicine.

The above-mentioned points have been updated in the Background and Discussion part in the revision.

**Q2.** A fundamental aspect of performing a benchmarking study is that it can be independently repeated by other researchers. Unfortunately this is not possible with the Quartet benchmark. There is no information how and where researchers can acquire the Quartet samples.

**Response:** Thank the reviewer for the professional comment. We apologize for not providing sufficient and complete Quartet samples information. We response this comment from the following five aspects: initiation and progression of the Quartet Project, establishment of the Quartet cell lines, cell culture and collection, preparation of the protein reference materials, and application of standard reference materials.

**1) About the initiation and progression of the Quartet Project.** This study was initiated by Fudan University in collaboration with over 30 institutions worldwide under the supervision and guidance of the National Institute of Metrology. As part of the Taizhou Longitudinal Study in Taizhou, Jiangsu, China, four healthy volunteers from a family Quartet, including father (F7), mother (M8), and monozygotic twin daughters (D5 and D6), were recruited and their peripheral blood was collected to establish the human immortalized cell lines. Large quantities of multi-omics reference materials suites (DNA, RNA, protein, and metabolite) were established simultaneously from these four immortalized cell lines. Each reference material was stored in over 1000 vials. They are applicable to a wide range of multi-omics technologies, including DNA sequencing, DNA methylation, RNAseq, miRNAseq, LC-MS/MS based proteomics, and metabolomics. This study was conducted in accordance with the principles of the Declaration of Helsinki and was approved by the Ethics Committee of the School of Life Sciences, Fudan University (BE2050). All four donors signed informed consent forms. As a part of the Quartet Project for the quality control and data integration of multi-omics profiling, the study of proteomic protein standard reference materials and datasets were uniformly deployed by Fudan University in 15 laboratories from six cities in China.

**2) About the establishment of the Quartet cell lines.** For establishing cell lines of genome, transcriptome, proteome, and metabolome reference materials, we adopted the same widely used protocol in the same laboratory of using Epstein-Barr virus (EBV) to establish immortalized lymphoblastoid cell lines (LCLs)[25] **(*Pharmacogenomics*, 2012, PMID: 22176622)**. Peripheral blood mononuclear cells (PBMCs) were isolated by Ficoll Lymphocyte Separation Solution. Naïve B cells were enriched by EasySep Human naïve B Cell Enrichment Kit (STEMCELL Technologies), and infected with Epstein-Barr virus (EBV) by centrifugation at 2000 rpm for 1 hour. After incubation, the successfully infected and immortalized cells were propagated in culture medium.

**3) About the cell culture** **and collection.** The Quartet cell lines were cultured in RPMI 1640 with 2 mM L-glutamine, 10% heat-inactivated FBS (fetal bovine serum), 1% penicillin, and 1% streptomycin (all from Sigma Aldrich, Gillingham, UK) at 37 °C with 5% CO2. The cells were passaged every 72 hours at a 1:4 split ratio. To obtain the multi-omics reference materials (DNA, RNA, protein, and metabolite), 2 × 10^9^ cells were cultured and harvested simultaneously for each type of cell line in each omics. Specifically, the cells grew in suspension and were centrifuged at 300 g for 5 minutes to obtain cell pellets. Mixed 2 × 10^9^ cells from the same type of cell line, collected them, and washed them twice with cold PBS. The cell pellets were stored at −80 °C until used as reference materials for the preparation of DNA, RNA, protein, and metabolite.

**4) About the preparation of the protein reference materials.** To obtain the protein reference materials, 2 × 10^9^ cells were collected from each cell line of each type, lysed in lysis buffer (8 M urea, 100 mM ammonium bicarbonate, pH 8.0) supplemented with protease inhibitors for 10 min on ice, and then sonicated for 3 min (2 s on and 2 s off) on ice and centrifuged at 16,000 × g for 20 min to remove the cell debris. The supernatants were collected, and the protein concentration was measured using a bicinchoninic acid protein assay. Extracted proteins were loaded into 10-kD Microcon filtration devices (Millipore, Burlington, MA), centrifuged at 16,000 × g for 20 min, and washed twice with urea lysis buffer and twice with 50 mM NH_4_HCO_3_. The samples were then digested using trypsin at an enzyme: protein mass ratio of 1:25 overnight at 37°C, after which peptides were extracted and dried (SpeedVac; Eppendorf, Hamburg, Germany). Two types of reference materials derived from 2 × 10^9^ cells of each of the four family members were uniformly prepared and sub-packed into 1,000 EP tubes with specific color labels (D5: blue, D6: green, F7: yellow, and M8: red) and stored in a -80℃ freezer. For generating the protein reference material, aliquots of 100 µg total protein were stored in each tube. For generating the peptide reference material, aliquots of 10 µg total tryptic peptide were stored in each tube. Before use, the protein reference materials were digested into peptides. We distributed these aliquots to 15 laboratories throughout six cities in China to participate in this testing (Fig. 1a). Subsequently, while applying for the First Class of National Reference Materials, the remaining two types of eight reference materials (four proteins and four peptides) have been uniformly transported to the National Institute of Metrology, providing convenience for laboratories around the world to obtain quality control materials through official channels.

**5) About the application of standard reference materials.** The Quartet Project provides the community with multi-omics (Genomic, transcriptomic, proteomic and Metabolomic) reference materials and reference datasets of four samples from a family Quartet, which are 1:1 matched, for objectively assessing quality in data generation and integrated analysis in increasingly large-scale multi-omics studies. It is worth mentioning that the DNA and RNA reference material suites have been approved by China's State Administration for Market Regulation as the First Class of National Reference Materials (Standard Material Numbers: GBW0900-GBW0907) and are extensively being utilized for proficiency testing and method validation. As a part of the Quartet Project for the quality control and data integration of multi-omics profiling, this Quartet Human B-lymphocyte proteomic protein standard reference materials are also in the national first-class reference materials approval process. After formal approval and release, the standard reference materials can not only be shared with various platforms, but also obtain standard reference datasets of materials through the “National Metrological Science Data Center” (https://srd.nmdc.ac.cn/gene/chinese-quartet/) for quality assessment between laboratories.

We have revised the information in the Methods part in the revision.

**Q3.** The Methods are lacking all but the most basic information, providing no details on what was done. What were the SOPs for the different instrument platforms? How was the bioinformatics analysis performed? For example, "Peptide quantification was performed according to the peak area," is the full extent to which label-free quantification is described, despite this being a major focus of the manuscript and advertised in the title. At the very least full information should be provided that allows other researchers to recreate this analysis, including software name, version, and detailed steps. Better would be if the analysis code was publicly available on e.g. GitHub.

**Response:** We thank the reviewer for the professional comments. To clearly response the question, we split it into four parts: LC-MS/MS analysis, peptide and protein identification, label-free-based MS quantification of proteins, and statistical analysis.

**1) LC-MS/MS analysis.** Samples were analyzed on Orbitrap Fusion, Orbitrap Fusion Lumos, Q Exactive, Q Exactive Plus, Q Exactive HF, Q-Exactive HF-X and Exploris 480 mass spectrometers (Thermo Fisher Scientific, Rockford, IL) coupled with a high-performance liquid chromatography system (EASY nLC 1000, Thermo Fisher Scientific), or a TripleTOF 6600+ (Sciex, Concord, Ontario) connected to Ultra Plus NanoLC 2D HPLC system (Eksigent Technologies, Dublin, CA), or a timsTOF Pro (Bruker Daltonics) set to acquire data in Parallel Accumulation Serial Fragmentation (PASEF) mode mass spectrometer connected to nanoElute (Bruker Daltonics). Dried peptide samples re-dissolved in Solvent A (0.1% formic acid in water) were loaded onto a 2-cm self-packed trap column (100 μm inner diameter, 3 μm ReproSil-Pur C18-AQ beads, Dr. Maisch GmbH) and separated on a 150-μm-inner-diameter column with a length of 15 cm (1.9 μm ReproSil-Pur C18-AQ beads, Dr. Maisch GmbH) over a 75-min gradient (Solvent A: 0.1% formic acid in water; Solvent B: 0.1% formic acid in 80% ACN) at a constant flow rate of 600 nL/min (0–75 min, 0 min, 4% B; 0–10 min, 4–15% B; 10–60 min, 15–30% B; 60–69 min, 30–50% B; 69–70 min, 50–100% B; 70–75 min, 100% B). **a) MS analysis (QE series).** Eluted peptides were ionized at 2 kV and introduced into the mass spectrometer. Mass spectrometry was performed in data-dependent acquisition mode. For the MS1 Spectra full scan, ions with m/z ranging from 300 to 1,400 were acquired by an Orbitrap mass analyzer at a high resolution of 120,000. The automatic gain control (AGC) target value was set to 3E+06. The maximal ion injection time was 80 ms. Top60 precursors were selected for MS2 experiment. The isolation window of selected precursor was 1.6 m/z. Precursor ions were fragmented with higher energy collision dissociation (HCD) with a normalized collision energy of 27 %. Fragment ions were analyzed by an Orbitrap mass analyzer with the resolution of 7,500, AGC target at 5E+04, as well as the maximum ion injection time of MS2 was 20 ms. Peptides that triggered MS/MS scans were dynamically excluded from further MS/MS scans for 12 s. **b) MS analysis (Fusion series).** Eluted peptides were ionized at 2 kV and introduced into the mass spectrometer. Mass spectrometry was performed in data-dependent acquisition mode. For the MS1 Spectra full scan, ions with m/z ranging from 300 to 1,400 were acquired by an Orbitrap mass analyzer at a high resolution of 120,000. The automatic gain control (AGC) target value was set to 3E+06. The maximal ion injection time was 80 ms. MS2 spectral acquisition was performed in the ion trap in a rapid speed mode with 1.5s cycletime. Precursor ions were selected and fragmented with higher energy collision dissociation (HCD) with a normalized collision energy of 27 %. Fragment ions were analyzed by an ion trap mass analyzer with an AGC target at 5E+04. The maximal ion injection time of MS2 was 20 ms. Peptides that triggered MS/MS scans were dynamically excluded from further MS/MS scans for 12 s. The coefficient of variation (CV) values on FAIMS were -45V and -65V. **c) MS analysis (TOF series).** For TripleTOF 6600+, Mass spectrometry was performed in data-dependent acquisition mode. MS1 spectra were collected between 300–1200 m/z for 250 ms. The 27 most intense precursor ions with charge states 2–4 that exceeded 300 counts per second were selected for fragmentation, and the corresponding fragmentation MS2 spectra were collected between 100–1700 m/z for 100 ms. After the fragmentation event, the precursor ions were dynamically excluded from reselection for 12 s. The mass spectrometer was a timsTOF Pro (Bruker Daltonics) set to acquire data in Parallel Accumulation Serial Fragmentation (PASEF) mode. The TIMS accumulation time was set to 100 ms and precursor masses for 0.4 min where charge states of 2-4 were allowed. The resolution parameter was set to 50,000 for MS1 and MS2. Mass spectra for MS1 and MS2 scans were recorded between 100 and 1700 m/z. Ion mobility resolution was set to 0.60–1.60 V·s/cm over a ramp time of 100 ms. Data-dependent acquisition (DDA) was performed using 10 PASEF MS/MS scans per cycle with a near 100% duty cycle. An active exclusion time of 0.4 min was applied to precursors that reached 20,000 intensity units.

**2) Peptide and protein identification.** MS raw files were processed using the “Firmiana”[26] **(*Nat. Biotechnol*, 2017, PMID: 28486446)** proteomics workstation. Briefly, raw files were searched against the NCBI human Refseq protein database using the Mascot search engine (Matrix Science Inc). The mass tolerances were: 20 ppm for precursor and 0.5 Da for product ions collected by Fusion series, 20 ppm for precursor and 50 mmu for product ions collected by QE series, 15 ppm for precursor and 0.05 Da for product ions collected by TOF series. Up to two missed cleavages were allowed. The database searching considered cysteine carbamidomethylation as a fixed modification, and N-acetylation, and oxidation of methionine as variable modifications. Precursor ion score charges were limited to +2, +3, and +4. For the quality control of protein identification, the target-decoy-based strategy was applied to confirm the FDR of both peptide and protein, which was lower than 1%. Percolator was used to obtain the quality value (q-value), validating the FDR (measured by the decoy hits) of every peptide-spectrum match (PSM), which was lower than 1%. Subsequently, all the peptides shorter than seven amino acids were removed. The cutoff ion score for peptide identification was 20. All the PSMs in all fractions were combined to comply with a stringent protein quality control strategy. We employed the parsimony principle and dynamically increased the q-values of both target and decoy peptide sequences until the corresponding protein FDR was <1%. Finally, to reduce the false positive rate, the proteins with at least one unique peptide were selected for further investigation[27, 28] **(*J Hematol Oncol*, 2012, PMID: 35659036; *Nat Commun*, 2022, PMID: 36347856)**.

**3) Label-free-based MS quantification of proteins.** Label-free-based MS quantification of proteins. The one-stop proteomic cloud platform “Firmiana”[26] **(*Nat. Biotechnol*, 2017, PMID: 28486446)** was further employed for protein quantification. Identification results and the raw data from the mzXML file were loaded. Then for each identified peptide, the extracted-ion chromatogram (XIC) was extracted by searching against the MS1 based on its identification information, and the abundance was estimated by calculating the area under the extracted XIC curve. For protein abundance calculation, the nonredundant peptide list was used to assemble proteins following the parsimony principle. The protein abundance was estimated using a traditional label-free, intensity-based absolute quantification (iBAQ) algorithm[29] **(*Nature*, 2011, PMID: 21593866)**, which divided the protein abundance (derived from identified peptides’ intensities) by the number of theoretically observable peptides. Subsequently, the fraction of total (FOT), a relative quantification value was defined as a protein’s iBAQ divided by the total iBAQ of all identified proteins in one experiment, and was calculated as the normalized abundance of a particular protein among experiments. Finally, the FOT values were further multiplied by 10^5^ for ease of presentation, and missing values were assigned 10^-5^[27, 28] **(*J Hematol Oncol*, 2012, PMID: 35659036; *Nat Commun*, 2022, PMID: 36347856)**.

**4) Statistical analysis.** Pearson’s correlation coefficient was calculated using the cor.test function in the R software. The protein overlap rate was used as a measure of reproducibility, and the CV was used as a measure of quantification consistency. For the heatmap, each protein expression value in the global proteomic expression matrix was transformed into a Z-score across all samples. For protein-wise clustering, the distance was set as “Euclidean’’ distance and the weight method was set to “complete.” The Z-score-transformed matrix was clustered using the R package pheatmap (version 1.0.12). The SNR was calculated from the inter- and intra-sample distances within principal component analysis dimensions **(Fig. 4e, upper panel)**. All other statistical analyses were performed with RGUI version 3.6, and publicly available on GitHub (https://github.com/ecnuzdd/MAQC/).

We have supplemented the full information in the Methods part in the revision.

**Q4.** MS/MS data is not available either. It is mentioned that it has been uploaded to iPROX, but the advertised dataset number does not exist or is possibly still private. In any case, I could not find the data.

**Response:** Thanks for the comments. We apologize for not providing sufficient and complete information for data acquisition. We response this comment from the following aspects:

**1) Study design and implementation.** The immortalized B-lymphoblastoid cell lines (LCLs) provided by a Chinese Quartet family from the Fudan Taizhou Cohort, consisting of father (F7), mother (M8), and monozygotic twin daughters (D5 and D6), were used to prepare the proteome reference materials (i.e., the Quartet) in both protein and peptide forms. The aliquots were sent to 15 laboratories, and proteomics analyses by data-dependent acquisition (DDA) was carried out using Orbitrap- and TOF-based systems. All LC-MS/MS files were aggregated at Fudan University for analysis.

**2) File description.** All measurements resulted in a global dataset containing 816 MS files generated from the 15 laboratories, providing a rich resource for performing both qualitative and quantitative reproducibility and reliability assessments while promoting the clinical translation and application of proteomics. Among the files, 792 MS files were used for the following purposes: cross-platform assessment with a single-shot proteomics strategy, which acquired a total of 288 LC-MS/MS runs (4 samples × 3 replicates × 24 experiments) using the Quartet standards at the peptide level; deep coverage with a sample fractionation strategy, which produced a total of 384 LC-MS/MS runs using the Quartet standards at the protein level; and longitudinal proteomic monitoring for 15 months, which was used for sample stability evaluation, generating 120 MS files using the Quartet standards at both peptide and protein levels.

**3)** **Data acquisition.** Raw files were deposited to iProX repository (<https://www.iprox.cn/>) with the accession number IPX0002859000. We have revised the access status, and you can access with URL: https://www.iprox.cn/page/SSV024.html;url=1682968594233IowD with the password 0V84. In addition, we updated the description in the part of “Data availability” in the revised manuscript.

**Q5.** Other experimental details are also missing or only vaguely described. For example, the SNR calculation is only detailed in Figure 4E, but is not even described in the Methods. Relevant related literature is again not described either, such as "QC metrics from CPTAC raw LC-MS/MS data interpreted through multivariate statistics" (http://pubs.acs.org/doi/abs/10.1021/ac4034455) which used PCA for quality control and to interpret sources of variability.

**Response:** Thank the reviewer for the professional comment. We apologize for not providing enough complete experimental details information. To clearly response the question, we split it into two parts.

**1) The SNR calculation.**

Signal-to-noise ratio (SNR) is a measurement used in science and engineering. SNR is defined as the ratio of the power of a signal to the power of noise and is often expressed in decibels (<https://en.wikipedia.org/wiki/Signal-to-noise_ratio>). In this study, the average distances representing the intrinsic "differences" among distinct biological sample groups are regarded as the signal, whereas the average distances among technical replicates of the same sample group are regarded as noise. To identify an effective way to characterize the SNR values, we evaluated the performances of SNR values calculated by different algorithms, including Euclidean distance (Dist), overall expression profiles (Expr), Pearson correlation coefficient (Cor), t-Distributed Stochastic Neighbor Embedding (tSNE), and principal components analysis (PCA), and found PCA based SNR outperformed. The numbers of PC components used in calculating SNR were then determined. We decided to use the first two components in PCA to calculate SNR values in correspondence with visualization in PCA plots.

Therefore, SNR is defined as E.q:

$$SNR=10\times{log}_{10}(\frac{m\times\binom{n}{2}}{\binom{m}{2}\times n\times n}\times\frac{\sum_{x=1}^{m-1} \sum_{y=x+1}^{m} \sum_{i=1}^{n} \sum_{j=1}^{n} \sum_{p=1}^{2} W_{p}(PC_{p,i,x}-PC_{p,j,y})^{2}}{\sum_{x=1}^{m} \sum_{i=1}^{n} \sum_{j=i+1}^{n} \sum_{p=1}^{2} W_{p}(PC_{p,i,x}-PC_{p,j,x})^{2}})$$

where 𝑚 is the number of sample groups, while 𝑛 is the number of replicates in each sample group. 𝑊_𝑝_ represents the *p*^th^ principal component of variances. 𝑃𝐶_𝑝,𝑖,𝑥_, 𝑃𝐶_𝑝,𝑗,𝑥_ and 𝑃𝐶_𝑝,𝑗,𝑦_ represent the *p*^th^ component values of replicate 𝑖 and replicate 𝑗 in sample group 𝑥 or sample group 𝑦, respectively.

A standard sample set consisted of 12 tubes with each representing one of the three replicates of the four protein reference materials. Therefore, a typical SNR in the study was the ratio of the average distances between different biological groups (9*12/2=54) to the average distances between technical replicates of the same groups (2*3*4/2=12). The distribution of intra-batch SNR values from 24 protein datasets was used to identify a threshold of 12 (mean-standard deviation), indicative of high discriminating power.

**2) Statistical analysis.** Pearson’s correlation coefficient (r) was calculated using the cor.test function in the R software. The protein overlap rate was used as a measure of reproducibility, and the CV was used as a measure of quantification consistency. For the heatmap, each protein expression value in the global proteomic expression matrix was transformed into a Z-score across all samples. For protein-wise clustering, the distance was set as “Euclidean’’ distance and the weight method was set to “complete.” The Z-score-transformed matrix was clustered using the R package pheatmap (version 1.0.12). The SNR was calculated from the inter- and intra-sample distances within principal component analysis dimensions. All other statistical analyses were performed with RGUI version 3.6, and publicly available on GitHub (https://github.com/ecnuzdd/MAQC/).

We have supplemented all the missing experimental details in the Methods part in the revision.

Reviewer #2 (Remarks to the Author):

The authors present the generation and proteomic characterization of a human standard reference material (referred to as Quartet) for the qualitative and quantitative evaluation of reproducibility and reliability of different MS-based proteomics strategies, instruments and laboratories. In addition, it enables the identification of potential variation sources and provides resources for development and optimization as well as they validated the stability of the material for at least one year.

The characterization of the Quartet is very well designed and included various parameters (e.g. identification numbers, intensity, gene ontology annotation, confidence intervals, fraction-of-total, signal-to-noise-ratio). The results are nicely presented and are very exciting and promising. The manuscript is well written with concise and understandable formulations. I only have a few suggestions/questions:

**Q1.** When comparing and discussing the evaluation of different MS instrument performances, maybe the authors could also mention and discuss the impact and significance of the coupled chromatographic system. To my understanding, these chromatographic systems were not uniform across the different laboratories. Thus, they definitely could influence the considered parameters of identification numbers, etc. and consequently additionally influence the overall performance, which makes a direct comparison of explicit MS instrument performances difficult.

**Response:** Thank the reviewer for the professional comment. We apologize for not providing sufficient and complete chromatographic systems information. To clearly response the question, we split it into two parts: the influence of liquid phase system on quantitative analysis in proteomics and the liquid phase system setup in our study.

**1) The influence of liquid phase system on quantitative analysis in proteomics**

The performance of the liquid phase system directly affects the separation and detection of proteins. For example, in a chromatography column, parameters such as the packing material, column length, diameter, flow rate, and chromatographic gradient directly affect the retention time, separation degree, and peak shape of the proteins. Therefore, for quantitative analysis in proteomics, the reasonable selection of liquid phase systems and optimization and calibration of system parameters are important steps to ensure the accuracy and reliability of the analysis results.

**2) The liquid phase system setup in our study**

Although different MS instruments are coupled with different chromatographic instruments, in order to maintain the consistency of the liquid phase system as much as possible, we have controlled the parameters of the chromatography column such as packing material, column length, diameter, flow rate, and chromatographic gradient. We also prepared chromatographic columns of the same length and diameter using the same packing material, distributed them to 15 laboratories, and set fixed ratios of mobile phase, flow rates, and chromatographic gradients.

In order to further evaluate the impact of different chromatographic instruments on proteome detection under the control of the above parameters, we compared two different types of chromatographic instruments. Taken Vanquish Neo and EASY nLC 1200, coupled to the same MS instrument (Orbitrap Ascend Tribrid; Thermo Fisher Scientific, Rockford, IL, USA) as examples, we tested D5 sample of the Quartet and the results showed that the total ion chromatogram (TIC) of the two MS detection results was basically consistent, and the retention time of several representative peptides was comparable (less than 1min of the front and back) **(Figure RL 7)**. Therefore, strict control of the above parameters of the liquid phase system can minimize the influence of different chromatographic instruments on the overall detection performance, thus making the performance of MS instruments comparable.

We have revised the information in the Discussion part in the revision.

**Figure RL 7** | Two different types of chromatographic instruments coupled with Ascend MS instrument for the detection of Quartet D5 samples.

**a,** The total ion chromatogram (TIC) of the detection results of two different types of chromatographic instruments. **b,** The RT of the peptide of two different types of chromatographic instruments.

**Q2.** In figure 1, the last subsection should be "C" instead of "D".

**Response:** Thanks for the comments. We apologize for our mistake. We have corrected "D" to "C" in the last subsection of figure 1, and checked all the annotations on the figures and tables to ensure they are correct.

**Q3.** Could the authors please mention how the used lymphoblastoid cells, used as basis for the generated Quartet material, were immortalized?

**Response:** Thank the reviewer for the professional comment. We apologize for not providing sufficient and complete Quartet samples information. We response this comment from the following three aspects: initiation and progression of the Quartet Project, establishment of the Quartet cell lines, and cell culture and collection.

**1) About the initiation and progression of the Quartet Project.** This study was initiated by Fudan University in collaboration with over 30 institutions worldwide under the supervision and guidance of the National Institute of Metrology. As part of the Taizhou Longitudinal Study in Taizhou, Jiangsu, China, four healthy volunteers from a family Quartet, including father (F7), mother (M8), and monozygotic twin daughters (D5 and D6), were recruited and their peripheral blood was collected to establish the human immortalized cell lines. Large quantities of multi-omics reference materials suites (DNA, RNA, protein, and metabolite) were established simultaneously from these four immortalized cell lines. Each reference material was stored in over 1000 vials. They are applicable to a wide range of multi-omics technologies, including DNA sequencing, DNA methylation, RNAseq, miRNAseq, LC-MS/MS based proteomics, and metabolomics. This study was conducted in accordance with the principles of the Declaration of Helsinki and was approved by the Ethics Committee of the School of Life Sciences, Fudan University (BE2050). All four donors signed informed consent forms. As a part of the Quartet Project for the quality control and data integration of multi-omics profiling, the study of proteomic protein standard reference materials and datasets were uniformly deployed by Fudan University in 15 laboratories from six cities in China.

**2) About the establishment of the Quartet cell lines.** For establishing cell lines of genome, transcriptome, proteome, and metabolome reference materials, we adopted the same widely used protocol in the same laboratory of using Epstein-Barr virus (EBV) to establish immortalized lymphoblastoid cell lines (LCLs)[25] **(*Pharmacogenomics*, 2012, PMID: 22176622)**. Peripheral blood mononuclear cells (PBMCs) were isolated by Ficoll Lymphocyte Separation Solution. Naïve B cells were enriched by EasySep Human naïve B Cell Enrichment Kit (STEMCELL Technologies), and infected with Epstein-Barr virus (EBV) by centrifugation at 2000 rpm for 1 hour. After incubation, the successfully infected and immortalized cells were propagated in culture medium.

**3) About the cell culture** **and collection.** The Quartet cell lines were cultured in RPMI 1640 with 2 mM L-glutamine, 10% heat-inactivated FBS (fetal bovine serum), 1% penicillin, and 1% streptomycin (all from Sigma Aldrich, Gillingham, UK) at 37 °C with 5% CO2. The cells were passaged every 72 hours at a 1:4 split ratio. To obtain the multi-omics reference materials (DNA, RNA, protein, and metabolite), 2 × 10^9^ cells were cultured and harvested simultaneously for each type of cell line in each omics. Specifically, the cells grew in suspension and were centrifuged at 300 g for 5 minutes to obtain cell pellets. Mixed 2 × 10^9^ cells from the same type of cell line, collected them, and washed them twice with cold PBS. The cell pellets were stored at −80 °C until used as reference materials for the preparation of DNA, RNA, protein, and metabolite.

We have supplemented all the missing experimental details in the Methods part in the revision.

**Q4.** Could the authors provide an outlook / test-case, how the Quartet could be used and integrated into potential translational/clinical studies as mentioned in the manuscript and how its usage could improve the studies´ quality?

**Response:** Thank the reviewer for the professional comment. To clearly response the question, we split it into two parts: preparation of the protein reference materials and application of standard reference materials.

**1) About the preparation of the protein reference materials.** To obtain the protein reference materials, 2 × 10^9^ cells were collected from each cell line of each type, lysed in lysis buffer (8 M urea, 100 mM ammonium bicarbonate, pH 8.0) supplemented with protease inhibitors for 10 min on ice, and then sonicated for 3 min (2 s on and 2 s off) on ice and centrifuged at 16,000 × g for 20 min to remove the cell debris. The supernatants were collected, and the protein concentration was measured using a bicinchoninic acid protein assay. Extracted proteins were loaded into 10-kD Microcon filtration devices (Millipore, Burlington, MA), centrifuged at 16,000 × g for 20 min, and washed twice with urea lysis buffer and twice with 50 mM NH_4_HCO_3_. The samples were then digested using trypsin at an enzyme: protein mass ratio of 1:25 overnight at 37°C, after which peptides were extracted and dried (SpeedVac; Eppendorf, Hamburg, Germany). Two types of reference materials derived from 2 × 10^9^ cells of each of the four family members were uniformly prepared and sub-packed into 1,000 EP tubes with specific color labels (D5: blue, D6: green, F7: yellow, and M8: red) and stored in a -80℃ freezer. For generating the protein reference material, aliquots of 100 µg total protein were stored in each tube. For generating the peptide reference material, aliquots of 10 µg total tryptic peptide were stored in each tube. Before use, the protein reference materials were digested into peptides. We distributed these aliquots to 15 laboratories throughout six cities in China to participate in this testing (Fig. 1a). Subsequently, while applying for the First Class of National Reference Materials, the remaining two types of eight reference materials (four proteins and four peptides) have been uniformly transported to the National Institute of Metrology, providing convenience for laboratories around the world to obtain quality control materials through official channels.

**2) About the application of standard reference materials.** The Quartet Project provides the community with multi-omics (Genomic, transcriptomic, proteomic and Metabolomic) reference materials and reference datasets of four samples from a family Quartet, which are 1:1 matched, for objectively assessing quality in data generation and integrated analysis in increasingly large-scale multi-omics studies. It is worth mentioning that the DNA and RNA reference material suites have been approved by China's State Administration for Market Regulation as the First Class of National Reference Materials (Standard Material Numbers: GBW0900-GBW0907) and are extensively being utilized for proficiency testing and method validation. As a part of the Quartet Project for the quality control and data integration of multi-omics profiling, this Quartet Human B-lymphocyte proteomic protein standard reference materials are also in the national first-class reference materials approval process. After formal approval and release, the standard reference materials can not only be shared with various platforms, but also obtain standard reference datasets of materials through the “National Metrological Science Data Center” (https://srd.nmdc.ac.cn/gene/chinese-quartet/) for quality assessment between laboratories.

The Quartet protein reference materials are established by complying with the ISO, and have been publicly available at chinese-quartet.org, as well as currently in the process of being approved. Our work is a crucial component of the MAQC-IV, and provides the life science community with and provides the life science community with a unique and valuable proteome resource, which could serve as a reference for the research community to evaluate new technologies, labs, assays, products, lab operators, and computational algorithms. Additionally, this work not only advances the field of proteomic quality control research based on previous work, but also contributes the proteomics power to conduct high-quality, large-scale multi-omics studies. We strongly recommend the use of Quartet reference materials to optimize experimental methods and monitor the status of experimental platforms for producing high-quality data, and discovering more useful knowledge, thereby promoting the development of precision medicine.

The above-mentioned points have been updated in the Methods and Discussion part in the revision.

**Q5.** The authors provide "anchor" proteins for potential calibration as well as "indicator" proteins to evaluate the quantitative reliability of different MS platforms. To corroborate their findings of anchor and indicator proteins, the authors could exemplarily validate the observed protein expression results with a complementary method, like targeted mass spectrometry or an antibody-dependent method.

**Response:** Thank the reviewer for the professional comment. To clearly response the question, we conducted PRM validation in reference materials.

The parallel reaction monitoring (PRM) assays are powerful targeted approaches to detect and quantify pre-specified proteins with a high throughput using high-resolution mass spectrometers. To further validate the "anchor" proteins for potential calibration as well as "indicator" proteins to evaluate the quantitative reliability of different MS platforms, we employed the targeted MS approach, PRM assays, to perform targeted assays on the Quartet samples (D5, D6, F7, and M8, × 3 repeats). Using search results from previous data, we directly selected a set of target peptides (Supplementary Table 7) that unique to "anchor" proteins (CARHSP1, CDK5RAP3, CHD4, DYNC1LI1,EIF4A2, ELF1, EML3, IRF4, IRF5, MAPK1, MAPK14, POLR2D, PURA, STAT1, STAT3) and designed the parallel reaction monitoring (PRM) method[28] **(*Nat Commun*, 2022, PMID: 36347856)**.

Peptide samples were injected into the Q Exactive HF-X Hybrid Quadrupole-Orbitrap Mass Spectrometer (Thermo Scientific) operating in PRM mode with quadrupole isolation and HCD fragmentation. The full MS mode was measured at resolution 60,000 with AGC target value of 3E6 and maximum IT of 20 ms, with scanning range of 300 to 1400 m/z. Target ions were submitted to MS/MS in the HCD cell (1.6m/z isolation width, 27% normalized collision energy). 15 PRM events were performed after MS1 scanning, at resolution 30,000 with AGC target value of 2E5 and maximum IT of 100 ms. Separation was achieved on a 150-μm-inner- diameter column with a length of 15 cm (1.9-μm ReproSil-Pur C18-AQ beads, Dr. Maisch GmbH) in an Easy 1200 nLC HPLC system (Thermo Scientific). Solvent A was 0.1 formic acid in MS-grade water and solvent B was 0.1% formic acid, 80% ACN in MS-grade water. Peptides were separated at 600 nL/min across a gradient ranging from 4 to 100% B over 75min (0–75 min, 0 min, 4% B; 0–10 min, 4–15% B; 10–60 min, 15–30% B; 60–69 min, 30–50% B; 69–70 min, 50–100% B; 70–75 min, 100% B).

Raw data was searched by Skyline-daily (4.2.1.19004, University of Washington, USA). The proteins were quantified with the fragment total area reported by Skyline-daily. We selected peptides and tested their stability of signal and shape of peaks in the pool sample for final quantification, and referred to the ranking offered by skyline.

Based on PRM quantification, we observed that these signature proteins can be detected in the Quartet samples, and the quantitative results show consistency within the same samples and heterogeneity between different samples **(Figure RL 8)**. In conclusion, our data demonstrated the stable expression of the "anchor" proteins were validated by PRM approach in the Quartet samples. Thus, the absolute quantification of the Quartet proteome potential calibration with "anchor" proteins have certain reliability, which also can provide reference datasets for QC and quality assessment of an LC-MS/MS platform for both basic research and clinical applications.

The above-mentioned points have been revised in the Methods and Results part in the revision.

**Figure RL 8** | Boxplot showing the expression of "anchor" proteins validated by PRM assay.

**References**

1.	Paulovich AG, Billheimer D, Ham AJ, Vega-Montoto L, Rudnick PA, Tabb DL, Wang P, Blackman RK, Bunk DM, Cardasis HL, et al: **Interlaboratory study characterizing a yeast performance standard for benchmarking LC-MS platform performance.** *Mol Cell Proteomics* 2010, **9:**242-254.

2.	Bell AW, Deutsch EW, Au CE, Kearney RE, Beavis R, Sechi S, Nilsson T, Bergeron JJ, Group HTSW: **A HUPO test sample study reveals common problems in mass spectrometry-based proteomics.** *Nat Methods* 2009, **6:**423-430.

3.	Tabb DL, Vega-Montoto L, Rudnick PA, Variyath AM, Ham AJ, Bunk DM, Kilpatrick LE, Billheimer DD, Blackman RK, Cardasis HL, et al: **Repeatability and reproducibility in proteomic identifications by liquid chromatography-tandem mass spectrometry.** *J Proteome Res* 2010, **9:**761-776.

4.	Campos A, Diaz R, Martinez-Bartolome S, Sierra J, Gallardo O, Sabido E, Lopez-Lucendo M, Ignacio Casal J, Pasquarello C, Scherl A, et al: **Multicenter experiment for quality control of peptide-centric LC-MS/MS analysis - A longitudinal performance assessment with nLC coupled to orbitrap MS analyzers.** *J Proteomics* 2015, **127:**264-274.

5.	Tabb DL, Wang X, Carr SA, Clauser KR, Mertins P, Chambers MC, Holman JD, Wang J, Zhang B, Zimmerman LJ, et al: **Reproducibility of Differential Proteomic Technologies in CPTAC Fractionated Xenografts.** *J Proteome Res* 2016, **15:**691-706.

6.	Bennett KL, Wang X, Bystrom CE, Chambers MC, Andacht TM, Dangott LJ, Elortza F, Leszyk J, Molina H, Moritz RL, et al: **The 2012/2013 ABRF Proteomic Research Group Study: Assessing Longitudinal Intralaboratory Variability in Routine Peptide Liquid Chromatography Tandem Mass Spectrometry Analyses.** *Mol Cell Proteomics* 2015, **14:**3299-3309.

7.	Amidan BG, Orton DJ, Lamarche BL, Monroe ME, Moore RJ, Venzin AM, Smith RD, Sego LH, Tardiff MF, Payne SH: **Signatures for mass spectrometry data quality.** *J Proteome Res* 2014, **13:**2215-2222.

8.	Collins BC, Hunter CL, Liu Y, Schilling B, Rosenberger G, Bader SL, Chan DW, Gibson BW, Gingras AC, Held JM, et al: **Multi-laboratory assessment of reproducibility, qualitative and quantitative performance of SWATH-mass spectrometry.** *Nat Commun* 2017, **8:**291.

9.	Bittremieux W, Walzer M, Tenzer S, Zhu W, Salek RM, Eisenacher M, Tabb DL: **The Human Proteome Organization-Proteomics Standards Initiative Quality Control Working Group: Making Quality Control More Accessible for Biological Mass Spectrometry.** *Anal Chem* 2017, **89:**4474-4479.

10.	Petralia F, Tignor N, Reva B, Koptyra M, Chowdhury S, Rykunov D, Krek A, Ma W, Zhu Y, Ji J, et al: **Integrated Proteogenomic Characterization across Major Histological Types of Pediatric Brain Cancer.** *Cell* 2020, **183:**1962-1985 e1931.

11.	Gao Q, Zhu H, Dong L, Shi W, Chen R, Song Z, Huang C, Li J, Dong X, Zhou Y, et al: **Integrated Proteogenomic Characterization of HBV-Related Hepatocellular Carcinoma.** *Cell* 2019, **179:**1240.

12.	Chen YJ, Roumeliotis TI, Chang YH, Chen CT, Han CL, Lin MH, Chen HW, Chang GC, Chang YL, Wu CT, et al: **Proteogenomics of Non-smoking Lung Cancer in East Asia Delineates Molecular Signatures of Pathogenesis and Progression.** *Cell* 2020, **182:**226-244 e217.

13.	Gillette MA, Satpathy S, Cao S, Dhanasekaran SM, Vasaikar SV, Krug K, Petralia F, Li Y, Liang WW, Reva B, et al: **Proteogenomic Characterization Reveals Therapeutic Vulnerabilities in Lung Adenocarcinoma.** *Cell* 2020, **182:**200-225 e235.

14.	Vasaikar S, Huang C, Wang X, Petyuk VA, Savage SR, Wen B, Dou Y, Zhang Y, Shi Z, Arshad OA, et al: **Proteogenomic Analysis of Human Colon Cancer Reveals New Therapeutic Opportunities.** *Cell* 2019, **177:**1035-1049 e1019.

15.	Zhang B, Wang J, Wang X, Zhu J, Liu Q, Shi Z, Chambers MC, Zimmerman LJ, Shaddox KF, Kim S, et al: **Proteogenomic characterization of human colon and rectal cancer.** *Nature* 2014, **513:**382-387.

16.	Clark DJ, Dhanasekaran SM, Petralia F, Pan J, Song X, Hu Y, da Veiga Leprevost F, Reva B, Lih TM, Chang HY, et al: **Integrated Proteogenomic Characterization of Clear Cell Renal Cell Carcinoma.** *Cell* 2019, **179:**964-983 e931.

17.	Qu Y, Feng J, Wu X, Bai L, Xu W, Zhu L, Liu Y, Xu F, Zhang X, Yang G, et al: **A proteogenomic analysis of clear cell renal cell carcinoma in a Chinese population.** *Nat Commun* 2022, **13:**2052.

18.	Dou Y, Kawaler EA, Cui Zhou D, Gritsenko MA, Huang C, Blumenberg L, Karpova A, Petyuk VA, Savage SR, Satpathy S, et al: **Proteogenomic Characterization of Endometrial Carcinoma.** *Cell* 2020, **180:**729-748 e726.

19.	Zhang H, Liu T, Zhang Z, Payne SH, Zhang B, McDermott JE, Zhou JY, Petyuk VA, Chen L, Ray D, et al: **Integrated Proteogenomic Characterization of Human High-Grade Serous Ovarian Cancer.** *Cell* 2016, **166:**755-765.

20.	Mertins P, Mani DR, Ruggles KV, Gillette MA, Clauser KR, Wang P, Wang X, Qiao JW, Cao S, Petralia F, et al: **Proteogenomics connects somatic mutations to signalling in breast cancer.** *Nature* 2016, **534:**55-62.

21.	Krug K, Jaehnig EJ, Satpathy S, Blumenberg L, Karpova A, Anurag M, Miles G, Mertins P, Geffen Y, Tang LC, et al: **Proteogenomic Landscape of Breast Cancer Tumorigenesis and Targeted Therapy.** *Cell* 2020, **183:**1436-1456 e1431.

22.	Liu Y, Mi Y, Mueller T, Kreibich S, Williams EG, Van Drogen A, Borel C, Frank M, Germain PL, Bludau I, et al: **Multi-omic measurements of heterogeneity in HeLa cells across laboratories.** *Nat Biotechnol* 2019, **37:**314-322.

23.	Liu Y, Beyer A, Aebersold R: **On the Dependency of Cellular Protein Levels on mRNA Abundance.** *Cell* 2016, **165:**535-550.

24.	Ding C, Li Y, Kim BJ, Malovannaya A, Jung SY, Wang Y, Qin J: **Quantitative analysis of cohesin complex stoichiometry and SMC3 modification-dependent protein interactions.** *J Proteome Res* 2011, **10:**3652-3659.

25.	Wheeler HE, Dolan ME: **Lymphoblastoid cell lines in pharmacogenomic discovery and clinical translation.** *Pharmacogenomics* 2012, **13:**55-70.

26.	Feng J, Ding C, Qiu N, Ni X, Zhan D, Liu W, Xia X, Li P, Lu B, Zhao Q, et al: **Firmiana: towards a one-stop proteomic cloud platform for data processing and analysis.** *Nat Biotechnol* 2017, **35:**409-412.

27.	Xu N, Yao Z, Shang G, Ye D, Wang H, Zhang H, Qu Y, Xu F, Wang Y, Qin Z, et al: **Integrated proteogenomic characterization of urothelial carcinoma of the bladder.** *J Hematol Oncol* 2022, **15:**76.

28.	Li Y, Xu C, Wang B, Xu F, Ma F, Qu Y, Jiang D, Li K, Feng J, Tian S, et al: **Author Correction: Proteomic characterization of gastric cancer response to chemotherapy and targeted therapy reveals potential therapeutic strategies.** *Nat Commun* 2022, **13:**6749.

29.	Schwanhausser B, Busse D, Li N, Dittmar G, Schuchhardt J, Wolf J, Chen W, Selbach M: **Global quantification of mammalian gene expression control.** *Nature* 2011, **473:**337-342.

**Second round of review**

**Reviewer 2**

all comments by both reviewers have been fully dressed; this will be a good resource!
